# Solvent-Free Synthesis, In Vitro and In Silico Studies of Novel Potential 1,3,4-Thiadiazole-Based Molecules against Microbial Pathogens

**DOI:** 10.3390/molecules27020342

**Published:** 2022-01-06

**Authors:** Ihsan A. Shehadi, Mohamad T. Abdelrahman, Mohamed Abdelraof, Huda R. M. Rashdan

**Affiliations:** 1 Pure and Applied Chemistry Research Group, Department of Chemistry, College of Sciences, University of Sharjah, Sharjah P.O. Box 27272, United Arab Emirates; ishehadi@sharjah.ac.ae; 2 Nuclear Research Centre, Radioisotopes Department, Egyptian Atomic Energy Authority, Cairo P.O. Box 12622, Egypt; medo2medo@gmail.com; 3 National Research Centre, Microbial Chemistry Department, Biotechnology Research Institute, 33 El Bohouth St. (Former El Tahrir St.), Giza P.O. Box 12622, Egypt; abdelraof87@gmail.com; 4 National Research Centre, Chemistry of Natural and Microbial Products Department, Pharmaceutical and Drug Industries Research Institute, Dokki, Cairo P.O. Box 12622, Egypt

**Keywords:** grindstone chemistry, 1,3,4-thiadiazoles, antimicrobial, MIC, molecular docking, molecular dynamics simulations

## Abstract

A new series of 1,3,4-thiadiazoles was synthesized by the reaction of methyl 2-(4-hydroxy-3-methoxybenzylidene) hydrazine-1-carbodithioate (**2**) with selected derivatives of hydrazonoyl halide by grinding method at room temperature. The chemical structures of the newly synthesized derivatives were resolved from correct spectral and microanalytical data. Moreover, all synthesized compounds were screened for their antimicrobial activities using *Escherichia coli*, *Pseudomonas aeruginosa*, *Proteus vulgaris*, *Bacillus subtilis*, *Staphylococcus aureus*, and *Candida albicans*. However, compounds **3** and **5** showed significant antimicrobial activity against all tested microorganisms. The other prepared compounds exhibited either only antimicrobial activity against Gram-positive bacteria like compounds **4** and **6**, or only antifungal activity like compound **7**. A molecular docking study of the compounds was performed against two important microbial enzymes: tyrosyl-tRNA synthetase (TyrRS) and N-myristoyl transferase (Nmt). The tested compounds showed variety in binding poses and interactions. However, compound **3** showed the best interactions in terms of number of hydrogen bonds, and the lowest affinity binding energy (−8.4 and −9.1 kcal/mol, respectively). From the in vitro and in silico studies, compound **3** is a good candidate for the next steps of the drug development process as an antimicrobial drug.

## 1. Introduction

**Figure 1:**
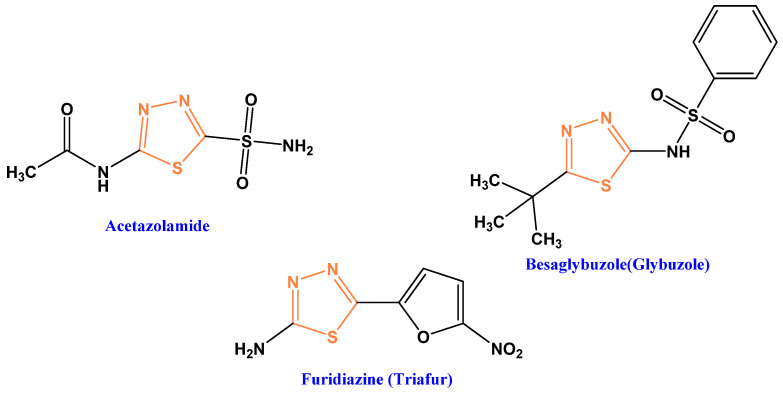
Chemical structures of drugs containing 1,3,4-thiadiazole moiety.

Microbial resistance toward most known classes of antibiotics is an acute problem, particularly in healthcare facilities. Several multidrug resistant pathogens such as *Staphylococcus aureus* (MRSA) show strong resistance against antibiotics containing lactams, glycopeptides, aminoglycosides, and fluoroquinolones. Furthermore, many studies indicated that extended spectrum β-lactamase-producing Enterobacteriaceae (ESBL-PE) and *Pseudomonas aeruginosa* can persist in the presence of several antibiotics through biofilm accumulation. In this way, the high resistance of different pathogens is increasingly identified as the cause for predominant causative pathogens in patients due to antibiotic misuse [1,2,3]. Consequently, the investigation of new heterocyclic compounds to encounter antibiotic resistance is among the most important aspects of preliminary pharmaceutical research. Consequently, a large number of studies should focus on producing new antibacterial drugs with entirely different chemical formulations and specific potential applications. Some compounds with the 1,3,4-thiadiazole framework emerged as potential pharmacophores in medicinal chemistry for their antimicrobial properties [4,5,6,7,8,9,10,11]. Many antibacterial and antiviral drugs were documented to append the 1,3,4-thiadiazole scaffold in their structures (Figure 1).

The 1,3,4-thiadiazoles were also involved in diverse biological applications [12,13,14,15,16] mostly by virtue of the –N double bond and C–S– group. Thiadiazole moieties can act as two-electron donor systems and hydrogen-binding domains. Moreover, 19 synthetic derivatives of 3,6-disubstituted-1,2,4-triazolo[3,4-*b*]-1,3,4-thiadiazole were screened for their activities against different microbial pathogens, and for antimicrobial activity. These compounds were superior to standard drugs such as ampicillin, streptomycin, and bifonazole. Hussein et al. (2008) [17] indicated significant antimicrobial activity of 1,3,4-thiadiazole derivatives against the *S. aureus* (Gram-positive) *E. coli* (Gram-negative) bacteria, and *A. niger* (multicellular fungi). A series of novel 1,3,4-thiadiazole derivatives containing an amide moiety were designed, prepared, and evaluated against bacterial rice diseases such as Xanthomonas oryzae pv. oryzae (Xoo) and Xanthomonas oryzae pv. oryzicola (Xoc) by Wu et al. (2021) [18]. The synthesized *N*-(5-benzylthio-1,3,4-thiadiazol-2-yl) and *N*-(5-benzylsulfonyl-1,3,4-thiadiazol-2-yl) derivatives of piperazinyl quinolones showed significant antibacterial activity against Gram-positive bacteria *S. aureus* and *S. epidermis* [19]. On the other hand, solvent-free organic synthesis is of great interest to introduce classical methods that are safer, cleaner, and easier to perform. Reactions without any solvent support can be employed with significant increases in reactivity and selectivity [2,20,21,22,23,24].

## 2. Results and Discussion

### 2.1. Chemistry

**Scheme 1:**
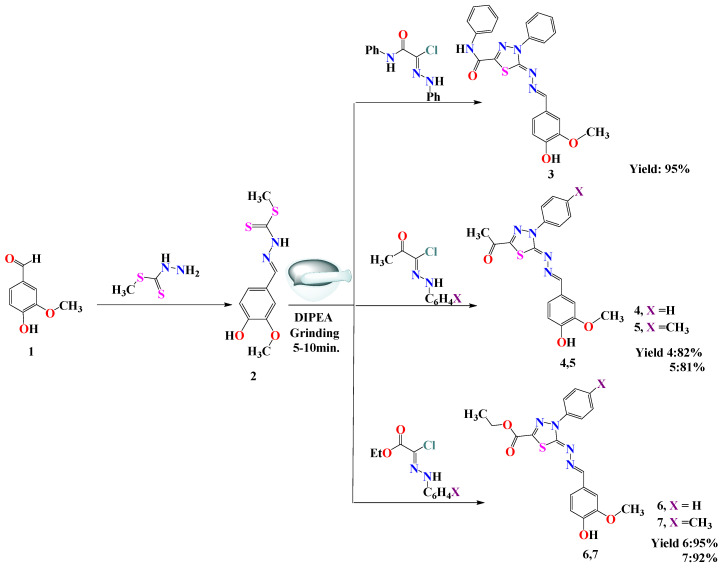
Solvent-free synthesis of 1,3,4-thiadiazole derivatives **3**–**7**.

Methyl 2-(4-hydroxy-3-methoxybenzylidene)hydrazine-1-carbodithioate (**2**) was reacted with a series of selected hydrazonoyl halide derivatives by a grinding method at room temperature in the presence of catalytic amounts of diisopropyl ethyl amine (2–3 drops) to give desired products **3**–**7** (Scheme 1). The chemical structures of synthesized derivatives **3**–**7** were inferred from their micro-analytical and spectral data. The IR spectrum of compound **3** showed a strong broad absorption band at *v* 3337 cm^−1^ for NH and OH groups. Compound **3** also exhibited a strong absorption band at *v* 1681 cm^−1^, which was attributed to the carbonyl group. The H1NMR spectrum of **3** showed a singlet signal at 3.83 ppm for methoxy group, and multiplet signals at 6.86–7.85 ppm for aromatic hydrogens. Compound **3** also showed a doublet signal at 7.75 ppm for aromatic hydrogen and doublet signal at 8.15 ppm for aromatic hydrogen. In addition, **3** exhibited three singlet signals at 8.36 and 9.65 ppm for CH–, OH and NH, respectively. Its C13NMR spectrum showed significant signals at *δ* 55.5, 110.05, 115.61, 120.96, 122.34, 122.43, 124.70, 125.58, 126.94, 128.82, 128.93, 137.54, 138.94, 147.33, 147.95, 149.54, 155.89, 156.26, and 164.16. The structure was also confirmed by its mass spectrum (*m*/*z* (445)) [M^+^], which agreed with its molecular formula, C_23_H_19_N_5_O_3_S. In addition, compound **4** revealed strong absorption bands at *v* 3417 and 1678 cm^−1^ for the OH and C=O groups, respectively; the H1NMR of **4** showed a singlet signal at δ 2.48 ppm for the methyl group, and a singlet signal at 3.80 ppm for the methoxy group. This is in addition to the doublet signals at 6.81 and 7.16 multiplet signals at 7.34–7.97 ppm for aromatic hydrogen besides the singlet signal at 8.31 for CH–, and the singlet signal at 9.65 for hydroxyl group. Its C13NMR exhibited characteristic signals at *δ* 25.13, 55.53, 110.01, 115.59, 122.60, 125.49, 127.42, 129.14, 138.53, 147.89, 149.55, 150.16, 156.32, and 164.03. The chemical structure of **4** was confirmed by the mass spectrum (*m*/*z* (%): 368 (M^+^), which agreed with the corresponding molecular formula. Moreover, compound **6** showed significant IR absorption bands at *v* 3471 and 1712 cm^−1^ for the hydroxyl and ester carbonyl groups, respectively. The H1NMR spectrum of **6** showed specific bands at the δ 1.29 triplet signal for the methyl group, a singlet signal at 3.82 for the methoxy group, a quartet signal at 4.15 for CH_2_, doublet signals at 6.83 and 7.17, multiplet signals at 7.33–7.45, a doublet signal at 7.90 for aromatic hydrogen, a singlet signal at 8.29 for CH–, and a singlet signal at 9.65 for hydroxyl group. C13NMR revealed characteristic signals at δ13.97, 55.54, 62.76, 110.19, 115.60, 122.41, 122.55, 125.50, 127.31, 127.90, 129.08, 138.60, 142.23, 147.91, 149.50, 156.11, 158.06, and 163.74 (details are presented in the experiments, and all data charts are attached as a Supplementary Materials).

### 2.2. Antimicrobial Screening

The rapid growth of multidrug resistance by microbial pathogens against major antibiotics is considered to be the most significant clinical problems facing the world [25]. The development of new compounds that could be derived from active groups to serve as wide-spectrum antimicrobials could contribute in combating such challenges. In the current study, the antimicrobial efficiency of seven synthetic 1,3,4-thiadiazole compounds against some pathogens was measured. Our results demonstrated that compounds **3** and **5** displayed wide-spectrum antimicrobial activity against all tested pathogens. The toxicity of compounds **4** and **6** toward Gram-positive bacteria was clearly noticed, and there was no activity observed against Gram-negative bacteria and yeast. Furthermore, compound **7** showed considerable activity against *C. albicans*, while it did not have any activity toward bacterial pathogens. In addition, there was no inhibition against all tested bacteria and *C. albicans* from compound **1**. The minimal inhibition concentration (MIC) of each compound was also determined, as shown in Table 1.

MIC is defined as the average of the lowest concentrations with no observable growth of microorganisms. Compound **3** exhibited considerable broad-spectrum antimicrobial activities against all strains of the tested pathogens, with low concentrations that were in the range of 20–40 µg/mL. Compound **5** provided an MIC value at low concentration toward Gram-positive bacteria, while it showed relatively higher MIC concentration against Gram-negative bacteria such as *Pseudomonas aeruginosa*. Even though compound **4** had an MIC value at low concentrations toward Gram-positive bacteria only, other tested organisms could be resistant to it even at high concentrations. In addition, MIC was observed at high concentration against *E. coli*, *S. aureus*, and *B. subtilis* for compound **6**. High resistance was also observed for all tested pathogens against compound **1**, while lower toxicity of compound **2** against *E. coli* was reported only with a high MIC value (320 µg/mL). The highest resistance of the bacterial models against these compounds, particularly Gram-negative bacteria, reflected a further mechanism to resisting toxic compounds that may be attributed to the cell wall in these bacteria. Previous studies demonstrated the antimicrobial activity of 1,3,4-thiadiazole derivatives, as they displayed potent antibacterial and antifungal activities [26].

### 2.3. Molecular Docking

Antibiotic resistance presents a great danger to human health. Tyrosyl-tRNA synthetase (TyrRS) is a member of aminoacyl tRNA synthetases that catalyze the attachment of amino acids to their corresponding tRNAs; therefore, they are indispensable for the synthesis of proteins. The similarity between bacterial and human TyRS qualifies TyRS as a good target for developing antibacterial drugs [27].

**Figure 2:**
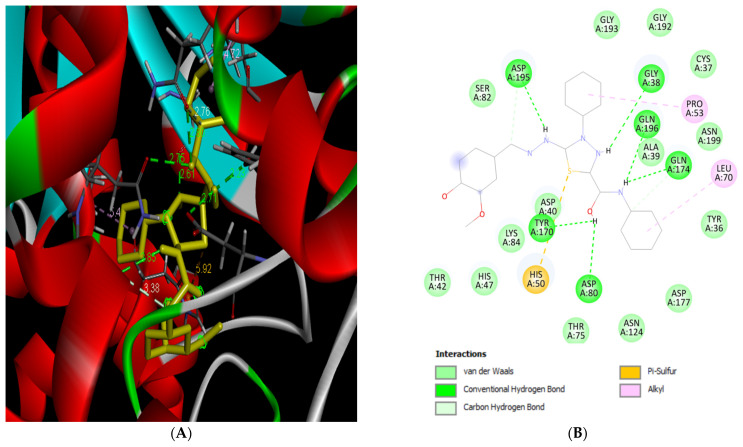
2D (**B**) and 3D (**A**) representation of compound **3** docking with TyrRS.

**Figure 3:**
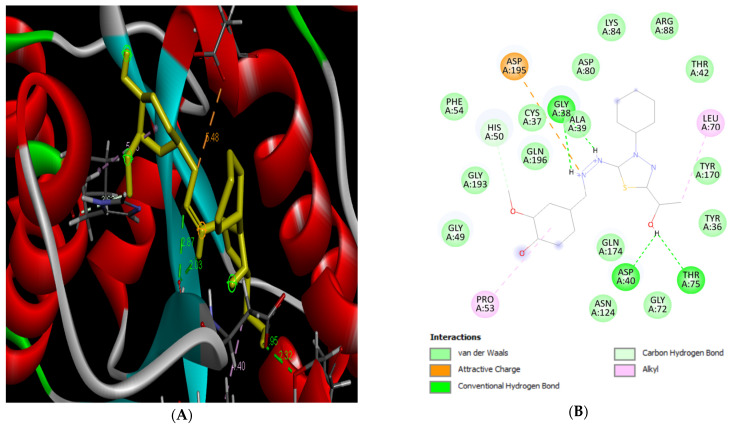
2D (**B**) and 3D (**A**) representation of compound **4** docking with TyrRS.

**Figure 4:**
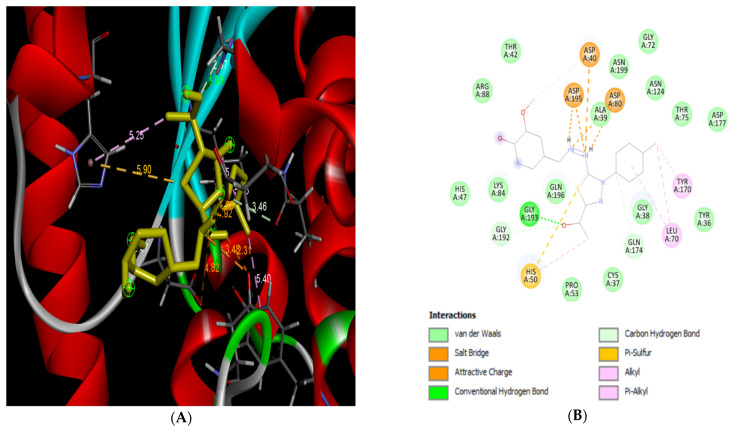
2D (**B**) and 3D (**A**) representation of compound **5** docking with TyrRS.

**Figure 5:**
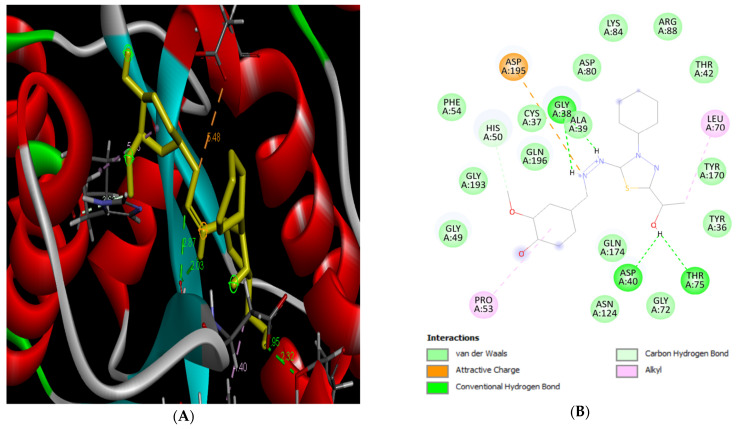
2D (**B**) and 3D (**A**) representation of compound **6** docking with TyrRS.

**Figure 6:**
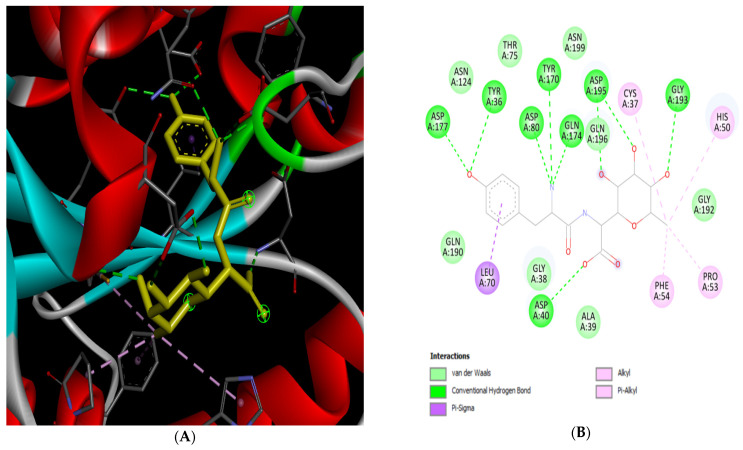
2D (**B**) and 3D (**A**) representation of TyrRS docked with its cocrystalized inhibitor.

**Figure 7:**
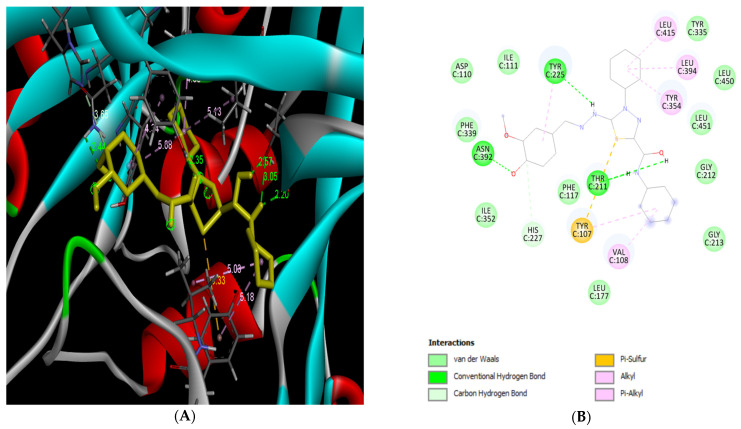
2D (**B**) and 3D (**A**) representation of compound **3** docking with Nmt.

**Figure 8:**
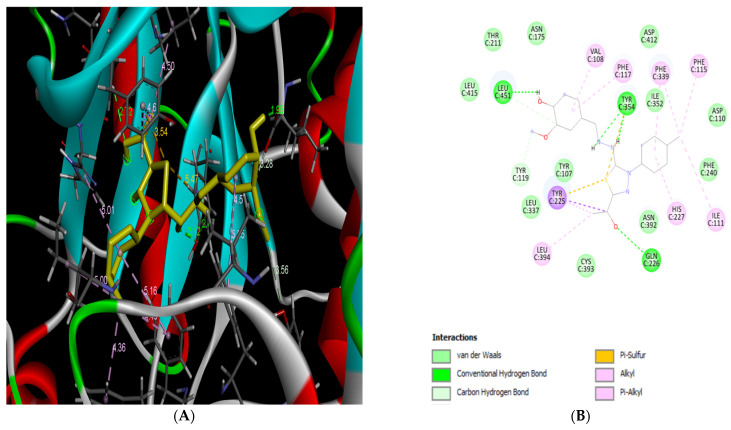
2D (**B**) and 3D (**A**) representation of compound **5** docking with Nmt.

**Figure 9:**
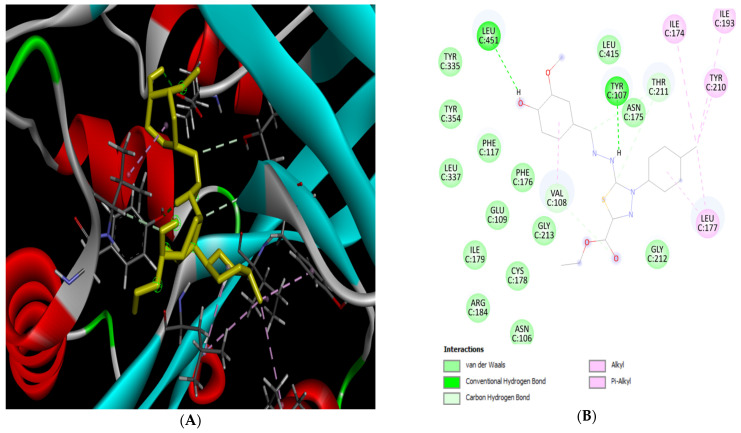
2D (**B**) and 3D (**A**) representation of compound **7** docking with Nmt.

**Figure 10:**
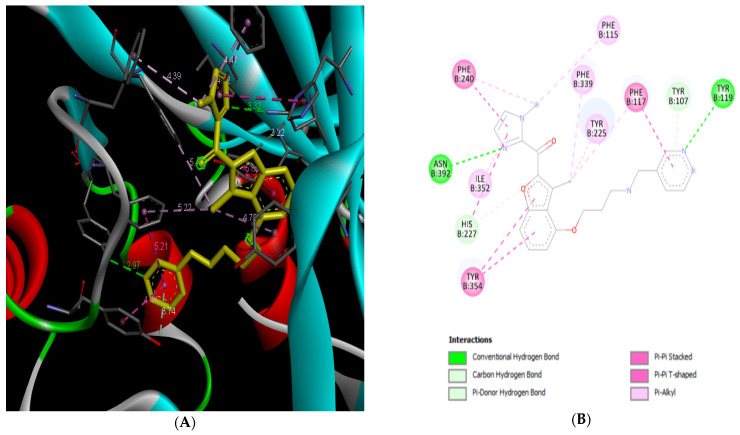
2D (**B**) and 3D (**A**) representation of Nmt docked with its cocrystalized inhibitor.

Compounds **3**–**6** showed different binding poses with *S. aureus* tyrosyl-tRNA synthetase (TyrRS), with a variety of interactions as summarized in Table 2 and Figure 2, Figure 3, Figure 4, Figure 5 and Figure 6. The binding and interactions between compounds **3**, **5**, and **7**, and *C. albicans* N-myristoyl transferase are shown in Table 3 and Figure 7, Figure 8, Figure 9 and Figure 10.

In the current study, the in vitro and molecular-docking results agreed. As shown in Table 2 and Figure 2, Figure 3, Figure 4, Figure 5 and Figure 6, compound **3** showed the best antibacterial activity, which agreed with its docking result with TyrRS. compound **3** was able to form 6 H bonds that were sufficient to stabilize the compound in the active site of the enzyme leading, to the inhibition and hindrance of the bacterial protein synthesis, instigating growth inhibition. Similarly, to the cocrystalized inhibitor in TyRS compound **3** could have formed H bonds with residues asp195, asp80, tyr170, and gln174, and interacted with other residues in sulfur and alky interactions like His50, leu70, and pro53. compound **3** excelled over its analog, compound **5**, in antibacterial activity due to the extra aniline group that was responsible for 2 more H bonds than compound **5** was (1 H bond at Gly193 and 2 nonclassical C–H bonds at asp40, gln174, and gly192). While the extra methyl group in compound **5** accounted for 3 alky interactions with tyr170 and leu70.

Compound **4** showed antibacterial activity due to its ability to form 4 H bonds with TyrRS cavity residues Gly38, thr75, gly38, and asp40. Only asp40 was similar to the cocrystalized inhibitor. On the other hand, compound **6** showed the least antibacterial activity compared to the **3**, **4**, and **5** derivatives. This could have been due to its failure to form any H bonds in TyrRS pocket, and it interacted only via alky and sulfur interactions with other residues such as His50, leu70, asp195, and pro53.

The transfer of the fatty acid myristate from myristoyl-CoA to the N-terminal glycine in several fungal and viral proteins is an important process. Such a transfer is catalyzed by N-myristoyltransferase (Nmt). Genetic and biochemical studies have recognized Nmt as a target for developing antifungal drugs. In the current study, compounds **3**, **5**, and **7** exhibited antifungal activity, with an advantage of compound **3** over the others. From the docking study with Nmt (Table 3 and Figure 7, Figure 8, Figure 9 and Figure 10), such an advantage could have been due to the potential ability of 3 to bind in the active site of Nmt causing its inhibition. compound **3** was able to form 5 H bonds that stabilized it in the pocket as an inhibitor. This action was similar to that of the cocrystalized inhibitor in the Nmt, where it could have formed hydrogen bond with the same residue, Asn392, in addition to 4 more H bonds with Thr211 (3H) and tyr225 within the pocket, with a number of possible interactions such as Pi–Pi in sulfur and alky groups as in His227, leu394, and leu415. Compounds 3 and 5, showed similar binding energies (−9.1 kcal/mol) to that of the cocrystalized inhibitor (−9.1 kcal/mol). However, compound **3** possessed an advantage over compounds **5** and **7** in terms of H bonds (5 versus 4 and 2) and another advantage over compound **7** in binding energy (−9.1 versus −8 kcal/mol) that facilitated the interaction for **7**.

From the wet lab and docking results, compounds **3** and **5** are recommended candidates for wide-spectrum antimicrobial agents, while compound **7** is recommended for more development as a specific antifungal agent. On the other hand, compounds **4** and **6** are recommended for further development as antibacterial agents.

### 2.4. Molecular Dynamics Simulations

**Figure 11:**
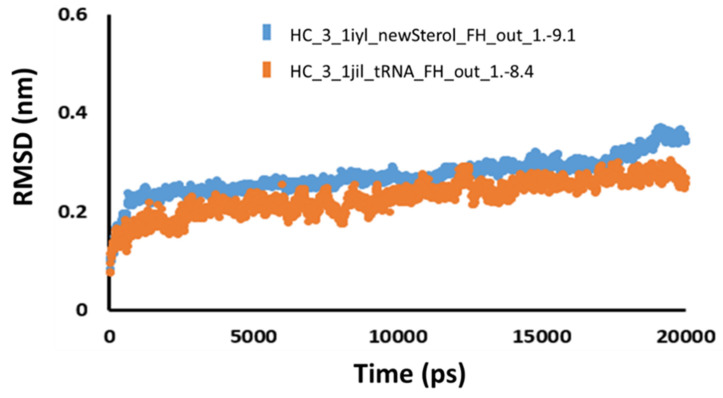
Root-mean-square deviation (RMSD) plot of both backbone proteins in simulated complex system with compound **3**.

Molecular dynamics (MD) simulations are extensively used to explore the stability and binding of various protein ligand complexes [28,29,30]. In order to elucidate the stability of complex structures, both proteins were bound with best binding compound **3** and subjected to MD simulations. The GROMACS 2019.2 software package was employed to perform MD simulations for the complexes with GROMOS force field force parameter set 54A7 [31]. Each protein complex was positioned in a cubic box, and periodic boundary conditions (PBCs) were applied. The simulated system was solvated with explicit simple point charge (SPC) model water molecules. To neutralize the systems, counter ions were added to the simulated complex systems. To calculate the electrostatic potential with PBCs, the particle-mesh Ewald (PME) method with a cutoff of 1.2 nm was applied. Bonds were constrained using the SHAKE algorithm. A steep descent energy minimization over 20,000 steps was followed by an equilibration in the NPT ensemble. Equilibration MD simulations were conducted using a Berendsen thermostat at 298.15 K with a temperature coupling constant of τ = 0.1 ps for 2 ns with a time step of 1.0 fs. A production run of 20 ns for each complex was conducted, and trajectories were prepared by saving conformations of the simulated systems for further analysis. Built-in modules g_rms and g_gyrate of GROMACS were used to conduct root-mean-square deviation (RMSD). RMSD analysis (Figure 11) showed that both proteins were stable throughout the simulations in both complex systems containing compound **3**.

## 3. Experimental Section

### 3.1. Chemistry

#### 3.1.1. Experimental Instrumentation

All melting points were determined on an electrothermal apparatus and were uncorrected. IR spectra were recorded (KBr discs) on a Shimadzu FT-IR 8201 PC spectrophotometer. H1NMR and C13NMR spectra were recorded in (CD_3_)_2_SO solutions on a BRUKER 500 FT-NMR system spectrometer, and chemical shifts were expressed in ppm units using TMS as an internal reference. Mass spectra were recorded on a GC–MS QP1000 EX Shimadzu. Elemental analyses were carried out at the microanalytical center of Cairo University.

#### 3.1.2. Synthesis

##### General Procedures for Synthesis of **3**–**7**

Methyl 2-(4-hydroxy-3-methoxybenzylidene)hydrazine-1-carbodithioate **2** (1.28 gm, 5 mmol) and the appropriate hydrazonoyl halides (5 mmol) with the addition of a few (2–3) drops of diisopropyl ethyl amine (DIPEA) were mixed and ground with a pestle in an open mortar at room temperature for 3–5 min till the mixture was turned into melt. The grinding of the initial syrup proceeded for 5–10 min, and the reaction was monitored by thin-layer chromatography (TLC). The obtained solid was washed with water and then ethanol, and eventually was recrystallized from ethanol to give target products **3**–**7**. 

*5-(-4-hydroxy-3-methoxybenzylidene)hydrazono)-N,4-diphenyl-4,5-dihydro-1,3,4-thiadiazole-2-carboxamide* (**3**). Yellow crystals, m.p. 251–253 °C; yield (95%); FT-IR (KBr, cm^−1^): *v* 3337 (broad band, NH, OH), 1681 (C=O), 1600 (C=N), 1539 (C=C); H1NMR (500 MHz, DMSO-*d*_6_): *δ* 3.83 (s, 3H, OCH_3_), 6.86–7.85 (m, 11H, ArH), 7.75 (d, 1H, *J* = 10 Hz, ArH), 8.15 (d, 1H, *J* = 10 Hz, ArH), 8.36 (s, 1H, CH), 9.65 (s, 1H, OH), 10.68 (s, 1H, NH); C13NMR (100 MHz, DMSO-*d*_6_): *δ* 55.5, 110.05, 115.61, 120.96, 122.34, 122.43, 124.70, 125.58, 126.94, 128.82, 128.93, 137.54, 138.94, 147.33, 147.95, 149.54, 155.89, 15626, 164.16; MS: *m*/*z* [%]: 445 (M^+^), 444 (75), 317 (30), 281 (28), 255 (80), 151 (52), 127 (48); Anal. Calcd. for C_23_H_19_N_5_O_3_S (445): C, 62.02; H, 4.30; N, 15.72% found: C, 62.08; H, 4.25; N, 15.69%.

*1-(5(-4-hydroxy-3-methoxybenzylidene)hydrazono)-4-phenyl-4,5-dihydro-1,3,4-thiadiazol-2-yl)ethan-1-one* (**4**). Orange solid (82%); mp. 212–214 °C, IR (KBr, cm^−1^): *v* 3417 (OH), 1678 (C=O), 1600 (C=N), 1550 (C=C); H1NMR (DMSO-*d*_6_): *δ* 2.48 (s, 3H, CH_3_), 3.80 (s, 3H, OCH_3_), 6.81 (d, 1H, *J* = 10 Hz, ArH), 7.16 (d, 1H, *J* = 10 Hz, ArH), 7.34–7.97 (m, 6H, ArH), 8.31 (s, 1H, CH), 9.65(s, 1H, OH); C13NMR (100 MHz, DMSO-*d*_6_): *δ* 25.13, 55.53, 110.01, 115.59, 122.60, 125.49, 127.42, 129.14, 138.53, 147.89, 149.55, 150.16, 156.32, 164.03; MS *m*/*z* (%): 368 (M^+^, 40). Anal. Calcd. for C_18_H_16_N_4_O_3_S (368): C, 58.68; H, 4.38; N, 15.21. Found: C, 58.62; H, 4.35; N, 15.18%.

*1-(5-(4-hydroxy-3-methoxybenzylidene)hydrazono)-4-(p-tolyl)-4,5-dihydro-1,3,4-thiadiazol-2-yl)ethan-1-one* (**5**). Orange crystals (81%); mp. 191–193 °C, IR (KBr, cm^−1^): *v* 3502 (OH), 1681 (C=O), 1600 (C=N), 1541 (C=C); H1NMR (DMSO-*d*_6_): *δ* 2.34 (s, 3H, CH_3_), 2.51 (s, 3H, CH_3_), 3.79 (s, 3H, OCH_3_), 6.80 (d, 1H, *J* = 10 Hz, ArH), 7.13 (d, 1H, *J* = 10 Hz), 7.30–7.32 (m, 3H, ArH), 7.80 (d, 2H, Ar–H), 8.27 (s, 1H, CH), 9.64 (s, 1H, OH); C13NMR (100 MHz, DMSO-*d*_6_): *δ* 20.65, 24.96, 55.51, 109.98, 115.57, 122.54, 125.52, 129.49, 136.21, 136.96, 147.93, 149.52, 149.94, 156.08, 164.12, 189.62; MS *m*/*z* (%): 382 (M^+^, 15)%. Anal. Calcd. for C_19_H_18_N_4_O_3_S (382): C, 59.67; H, 4.74; N, 14.65. Found: C, 59.72; H, 4.71; N, 14.61%.

*Ethyl 5-((-4-hydroxy-3-methoxybenzylidene)hydrazono)-4-phenyl-4,5-dihydro-1,3,4-thiadiazole-2-carboxylate* (**6**). Yellow crystals (95%); m.p. 172–174 °C, IR (KBr, cm^−1^): *v* 3471 (OH), 1712 (C=O, ester carbonyl), 1599 (C=N), 1554 (C=C); H1NMR (DMSO-*d*_6_): *δ* 1.29 (t, 3H, CH_3_), 3.82 (s, 3H, OCH_3_), 4.15 (q, 2H, CH_2_), 6.83 (d, 1H, *J* = 10 Hz, ArH), 7.17 (d, 1H, *J* = 10 Hz, ArH), 7.33–7.45 (m, 4H, Ar–H), 7.90 (d, 2H, *J* = 10 Hz, ArH), 8.29 (s, 1H, CH), 9.65 (s, 1H, OH); C13NMR (100 MHz, DMSO-*d*_6_): *δ* 13.97, 55.54, 62.76, 110.19, 115.60, 122.41, 122.55, 125.50, 127.31, 127.90, 129.08, 138.60, 142.23, 147.91, 149.50, 156.11, 158.06, 163.74; MS *m*/*z* (%): 398(M^+^, 80). Anal. Calcd. for C_19_H_18_N_4_O_4_S (398): C, 57.28; H, 4.55; N, 14.06. Found: C, 57.32; H, 4.52; N, 14.02%.

*Ethyl 5-((-4-hydroxy-3-methoxybenzylidene)hydrazono)-4-(p-tolyl)-4,5-dihydro-1,3,4-thiadiazole-2-carboxylate* (**7**). Yellow crystals (92%); m.p. 180–182 °C, IR (KBr, cm^−1^): *v* 3502 (OH), 1705 (C=O, carbonyl ester), 1600 (C=N), 1550 (C=N); H1NMR (DMSO-*d*_6_): *δ* 1.30(t, 3H, CH_3_), 2.36 (s, 3H, CH_3_), 3.81 (s, 3H, OCH_3_), 4.33 (q, 2H, CH_2_), 6.84(d, 1H, *J* = 10 Hz, ArH), 7.18 (d, 1H, *J* = 10 Hz, ArH), 7.31–7.33 (m, 3H, Ar–H), 7.76(d, 2H, *J* = 10 Hz, ArH), 8.29 (s, 1H, CH), 9.65(s, 1H, OH); C13NMR (100 MHz, DMSO-*d*_6_): *δ* 13.97, 20.63, 55.54, 62.71, 110.19, 115.59, 122.34, 122.55, 125.50, 129.46, 136.09, 136.90, 141.89, 147.90, 149.55, 155.93, 158.07, 163.80; MS *m*/*z* (%):412(M^+^, 30). Anal. Calcd. for C_20_H_20_N_4_O_4_S (412): C, 58.24; H, 4.89; N, 13.58. Found: C, 58.28; H, 4.81; N, 13.55%.

### 3.2. Antimicrobial Activity of Thiadiazole Derivatives

Antimicrobial susceptibility and minimal inhibitory concentration (MIC) of the synthesized thiadiazoles were determined toward three Gram-negative bacteria (*Escherichia coli* ATCC 25955, *Pseudomonas aeruginosa* ATCC 10145, and *Proteus vulgaris*), two Gram-positive bacteria (*Bacillus subtilis* ATCC 6633 and *Staphylococcus aureus* NRRL B-767), and unicellular fungi (*Candida albicans* ATCC 10231). The pathogens under study were provided by Al-Azhar University, Faculty of Medicine, Microbiology and Immunology Department, Egypt. The microbial pathogens were cultivated in Mueller Hinton broth at 35 ± 2 °C for 24 h. Antimicrobial activity and MIC were established a described by El-Bendary et al., 2020; Qader et al., 2021 [32,33]. Initially, thiadiazole compounds were screened for their ability to inhibit microbial growth by the agar well method. Subsequently, MIC was determined for the tested compounds using 96-well microplates (GAMA GROUP, Czech Republic) by transferring 10 µL of bacterial or fungal cells at the log phase to 180 µL of Mueller Hinton broth. Then, 10 µL of the tested thiadiazole compounds was added with different concentrations of 5, 10, 20, 40, 80, 160, and 320 µg/mL to the plates, which were then incubated at 35 ± 2 °C for 24 h. Lastly, the treated microorganisms were inhibited by measuring absorbance at 600 nm and compared with the controls (microorganisms without treatment) using a Spectrostar Nano Microplate Reader (BMG LABTECH GmbH, Allmendgrun, Germany). The percentage inhibition was calculated as [(A − B)/A] × 100, where A and B are the OD600 of microorganisms that were grown in the absence and presence of thiadiazole compounds, respectively. Obtained results were compared with reference antibiotic ciprofloxacin for bacterial cultures, and nystatin for *C. albicans* to evaluate the potency of the tested compounds under the same conditions [34].

### 3.3. Molecular Docking

The crystal structures of *Staphylococcus aureus* tyrosyl-tRNA synthetase (TyrRS) and *Candida albicans* N-myristoyl transferase (Nmt) were downloaded from a protein databank via http://www.rcsb.org (accessed on 24 November 2021). (PDB id: 1jil and1iyl) to be docked against the synthesized compounds. The target proteins were optimized for docking by removing water molecules, the cocrystalized inhibitors (SB284485 [2-amino-3-(4-hydroxy-phenyl)-propionylamino]-(3,4,5-trihydroxy-6-methyl-tetrahydro-pyran-2-YL)-acetic acid and (1-methyl-1H-imidazol-2-yl)-(3-methyl-4-{3-[(pyridine-3-yl methyl)-amino]-proxy}-benzofuran-2-YL)-methanone) respectively), and lastly adding hydrogen; then, they were saved as PDB files using Biovia Discovery Studio 2021. Compounds **3**–**6** were docked against “1jil” with cavity space dimensions x: 35.400, y: 16.615, and z: 82.825, while compounds **3**, **5** and **7** were docked against “1iyl” with cavity space dimensions x: 30.597, y: −9.247, and z: −25.658. Docking was performed using the CB-Dock web server (http://clab.labshare.cn/cb-dock/php/) (accessed on 24 November 2021) following its standard protocol [35].

## 4. Conclusions

Utilizing vanillin, methyl hydrazine carbidithioate, and appropriate hydrazonoyl halides, a series of 1,3,4-thiadiazole-based molecules were synthesized, and their chemical structures were inferred from their correct spectral and microanalytical data. Upon evaluation of the antimicrobial activities of these compounds using molecular docking, molecular dynamic simulations, and biological essays, it was proven that compounds **3** and **5** displayed high activity against all tested microorganisms, which were close to standard drugs (ciprofloxacin and nystatin); thus, they are potential novel antimicrobial drugs.

## Figures and Tables

**Table 1:** Antimicrobial activity and minimal inhibitory concentration (MIC) of synthesized thiadiazole derivatives.

Sample No.	Minimal Inhibitory Concentration (MIC, µg/mL)					
	*Escherichia coli*	*Pseudomonas aeruginosa*	*Proteus vulgaris*	*Bacillus subtilis*	*Staphylococcus aureus*	*Candida albicans*
1	ND	ND	ND	ND	ND	ND
2	320	ND	ND	ND	ND	ND
3	20	40	20	10	20	20
4	ND	ND	ND	40	80	ND
5	40	160	80	40	20	20
6	160	ND	ND	80	160	ND
7	ND	ND	ND	ND	ND	80
Ciprofloxacin	5	7	1.25	2.5	1.25	ND
Nystatin	ND	ND	ND	ND	ND	5

Ciprofloxacin and nystatin were used as positive controls; ND: not determined.

**Table 2:** Docking results of new compounds with TyrRS.

Entry	Binding Energy (kcal/mol)	H-Bonds		Residual Interactions
		Number	Residues	
**3**	−8.4	6	Asp195, gly196, asp80, gly38, tyr170, gln174	His50, leu70, asp195, pro53
**4**	−8.4	4	Gly38, thr75, gly38, asp40	His50, leu70, asp195, pro53
**5**	−8.4	1	Gly193	His50, leu70, asp195, asp80, tyr170, gln174, asp40, gly192
**6**	−7.7	0	NA	His50, leu70, asp195, pro53 asp80, cys37, tyr170, tyr36 Ala39, asp40, gln174
**Cocrystalized inhibitor**	−9.7	9	Asp195, gly193, asp80, asp40, tyr170, gln174, tyr36, asp177	His50, leu70, cys37, pro53

**Table 3:** Docking results of new compounds with N-myristoyl transferase (Nmt).

Entry	Binding Energy (kcal/mol)	H-Bonds		Residual Interactions
		Number	Residues	
**3**	−9.1	5	Thr211, tyr225, thr211, asn392, thr211	His227, leu394, leu415, val108, tyr107
**5**	−9.1	4	Tyr354, leu451, Tyr354, gln226	His227, leu394, ile111, val108, tyr119, phe117, phe115
**7**	−8.0	2	Tyr107, leu451	Thr211, val108, leu177, tyr210, ile193, ile174,
**Cocrystalized inhibitor**	−10	2	Asn392, tyr119	His227, phe117, Tyr354, phe240, phe115, ile352, tyr225, phe339

## Data Availability

Not applicable.

## Supplementary Materials

The following supporting information can be downloaded, Figure S1: H1NMR spectrum of comp. **3**, Figure S2: Magnification of H1NMR spectrum of comp. **3**, Figure S3: Magnification of H1NMR spectrum of comp. **3**, Figure S4: C13NMR spectrum of comp. **3**, Figure S5: H1NMR spectrum of comp. **4**, Figure S6: Magnification of H1NMR spectrum of comp. **4**, Figure S7: Magnification of H1NMR spectrum of comp. **4**, Figure S8: C13NMR spectrum of comp. **4**, Figure S9: H1NMR spectrum of comp. **5**, Figure S10: Magnification of H1NMR spectrum of comp. **5**, Figure S11: Magnification of H1NMR spectrum of comp. **5**, Figure S12: Magnification of H1NMR spectrum of comp. **5**, Figure S13: C13NMR spectrum of comp. **5**, Figure S14: H1NMR spectrum of comp. **6**, Figure S15: Magnification of H1NMR spectrum of comp. **6**, Figure S16: Magnification of H1NMR spectrum of comp. **6**, Figure S17: Magnification of H1NMR spectrum of comp. **6**, Figure S18: C13NMR spectrum of comp. **6**, Figure S19: H1NMR spectrum of comp. **7**, Figure S20: Magnification of H1NMR spectrum of comp. **7**, Figure S21: Magnification of H1NMR spectrum of comp. **7**, Figure S22: Magnification of H1NMR spectrum of comp. **7**, Figure S23: C13NMR spectrum of comp. **7**, Figure S24: IR of comp. **3**, Figure S25: IR of comp. **4**, Figure S26: IR spectrum of comp. **5**, Figure S27: IR of comp. **6**, Figure S28: IR of comp. **7**, Figure S29: Mass Spectrum of comp. **3**.

## Sample Availability

Samples of the compounds **3**–**7** are available from the authors.
