# Supplementary material for: Solvent-Free Synthesis, In Vitro and In Silico Studies of Novel Potential 1,3,4-Thiadiazole-Based Molecules against Microbial Pathogens
Source: Molecules. 2022 Jan 6;27(2):342. doi: 10.3390/molecules27020342 (PMC8779762; doi:10.3390/molecules27020342)
Supplement: Supplementary file 1 [file molecules-27-00342-s001.zip › molecules-1503696-supplementary.pdf]

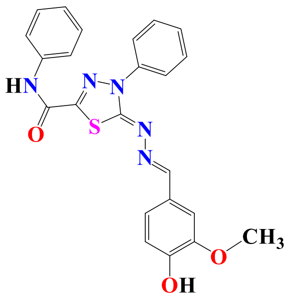

3

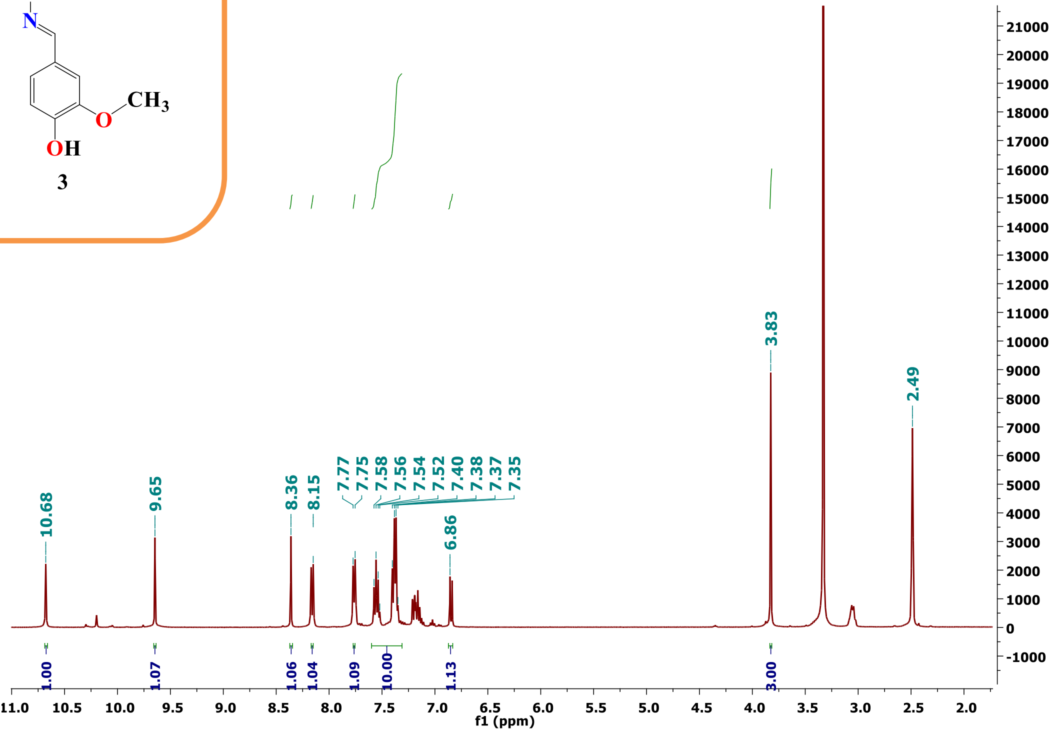

Figure S1: H1NMR spectrum of comp. **3**.

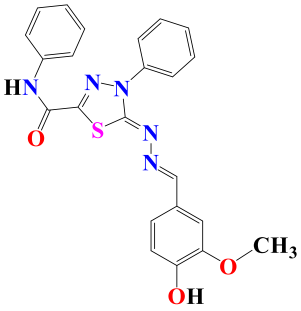

3

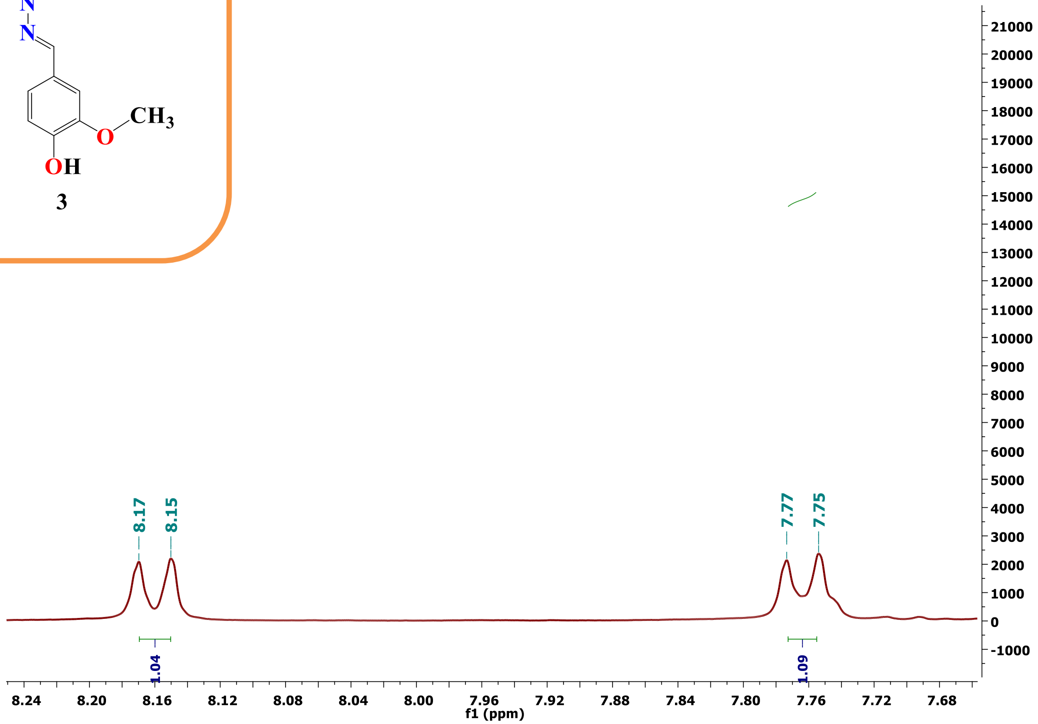

Figure S2: Magnification of H1NMR spectrum of comp. **3**.

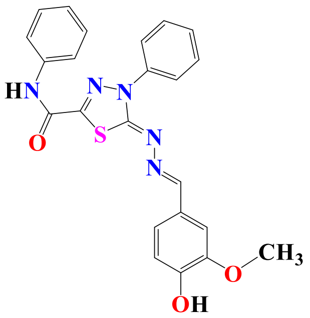

3

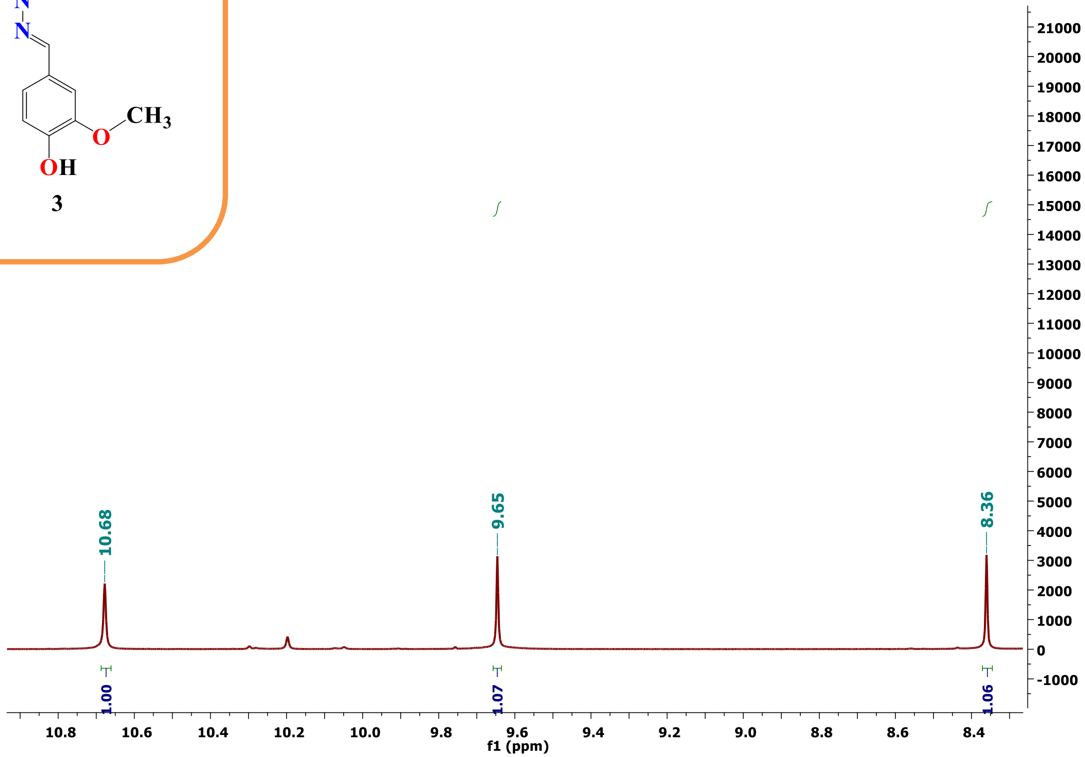

Figure S3: Magnification of H1NMR spectrum of comp. **3**.

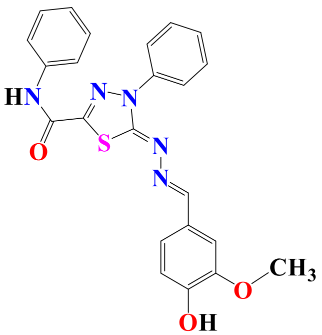

3

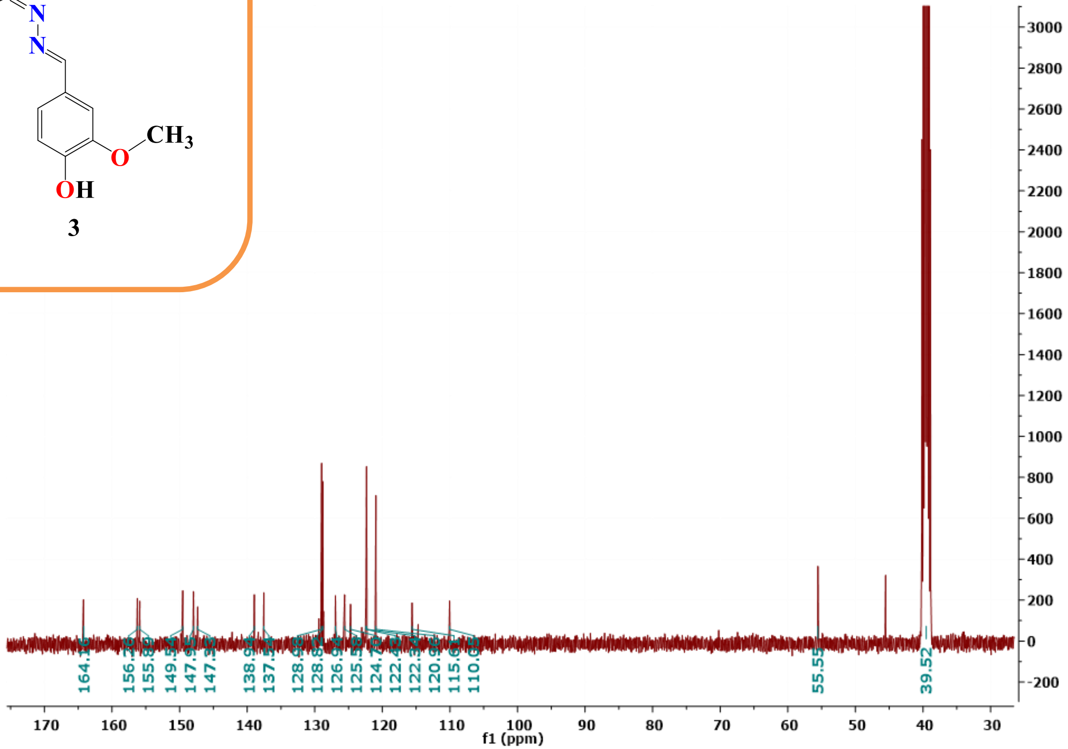

Figure S4: C13NMR spectrum of comp. **3**.

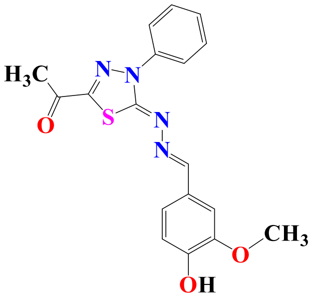

4

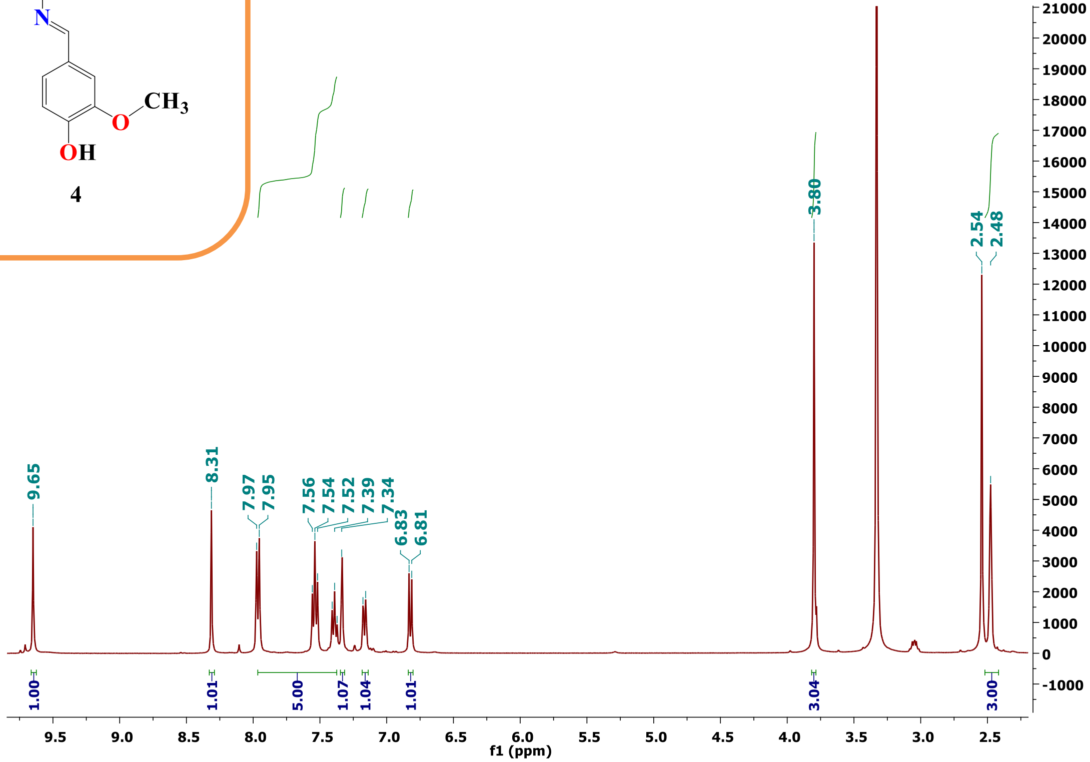

Figure S5: H1NMR spectrum of comp. **4**.

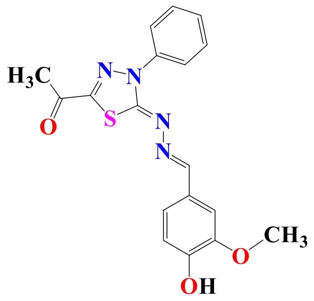

4

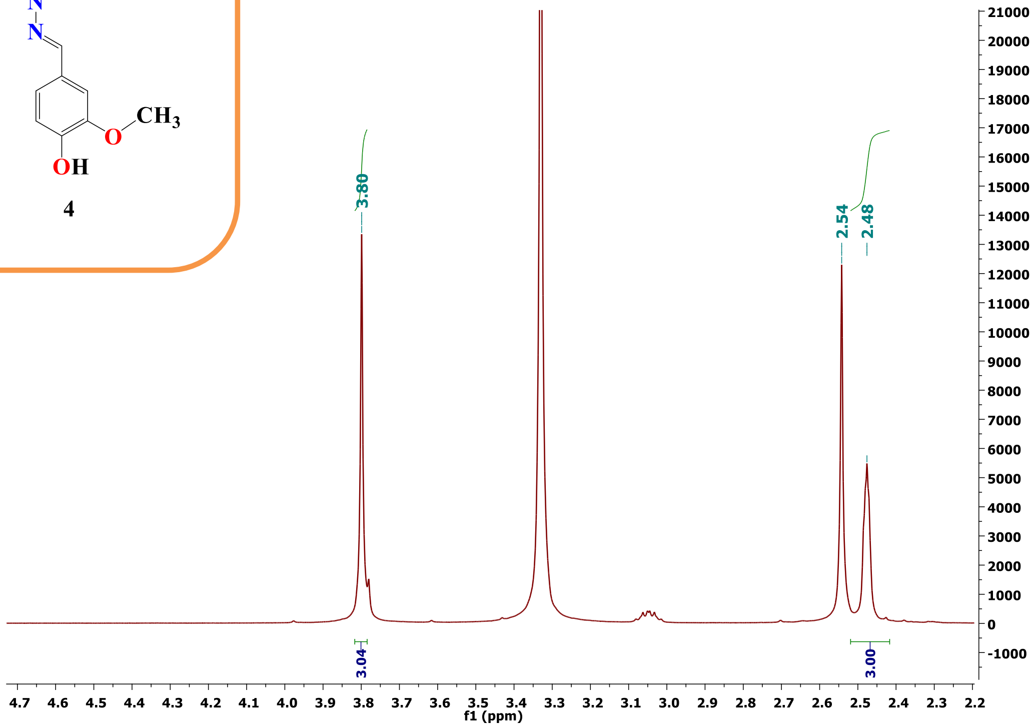

Figure S6: Magnification of H1NMR spectrum of comp. **4**.

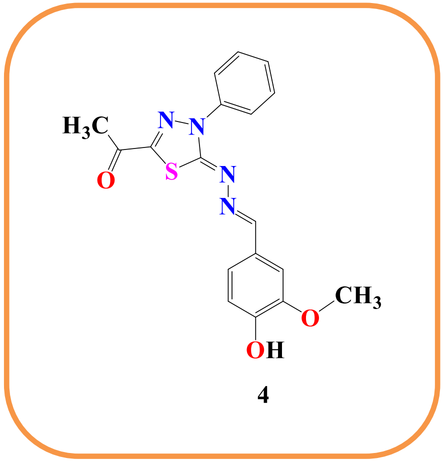

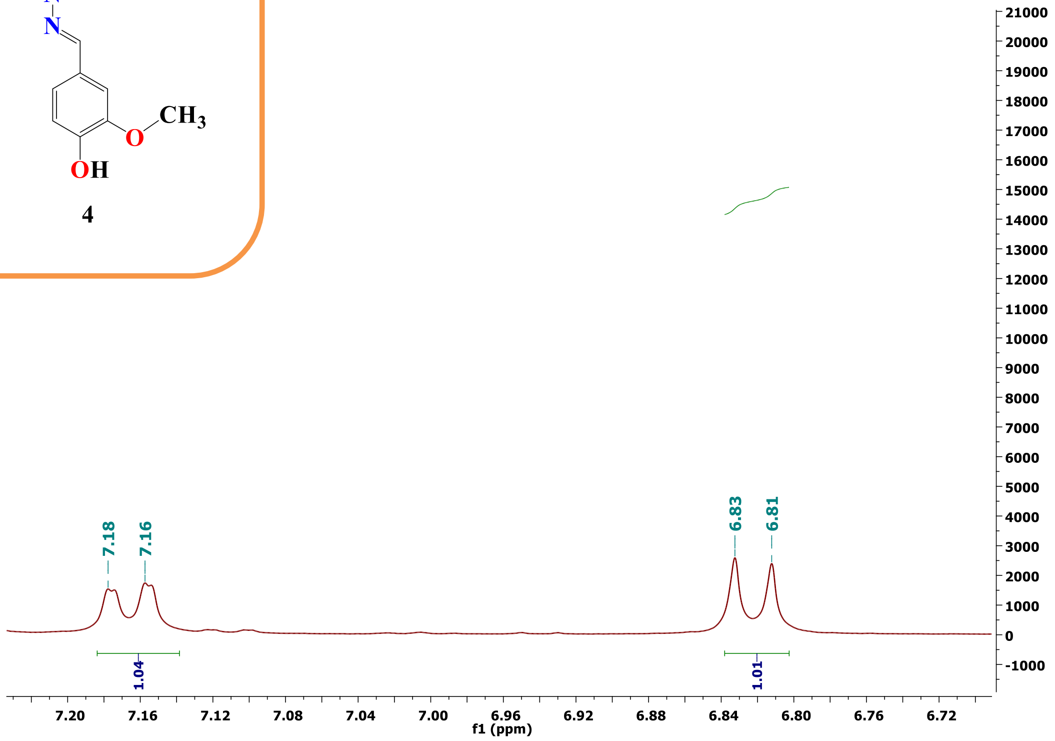

Figure S7: Magnification of H1NMR spectrum of comp. **4**.

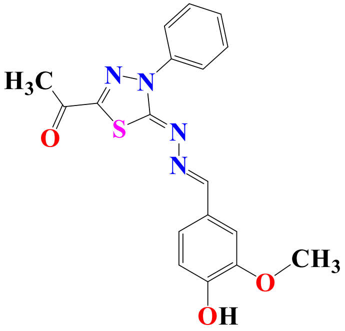

4

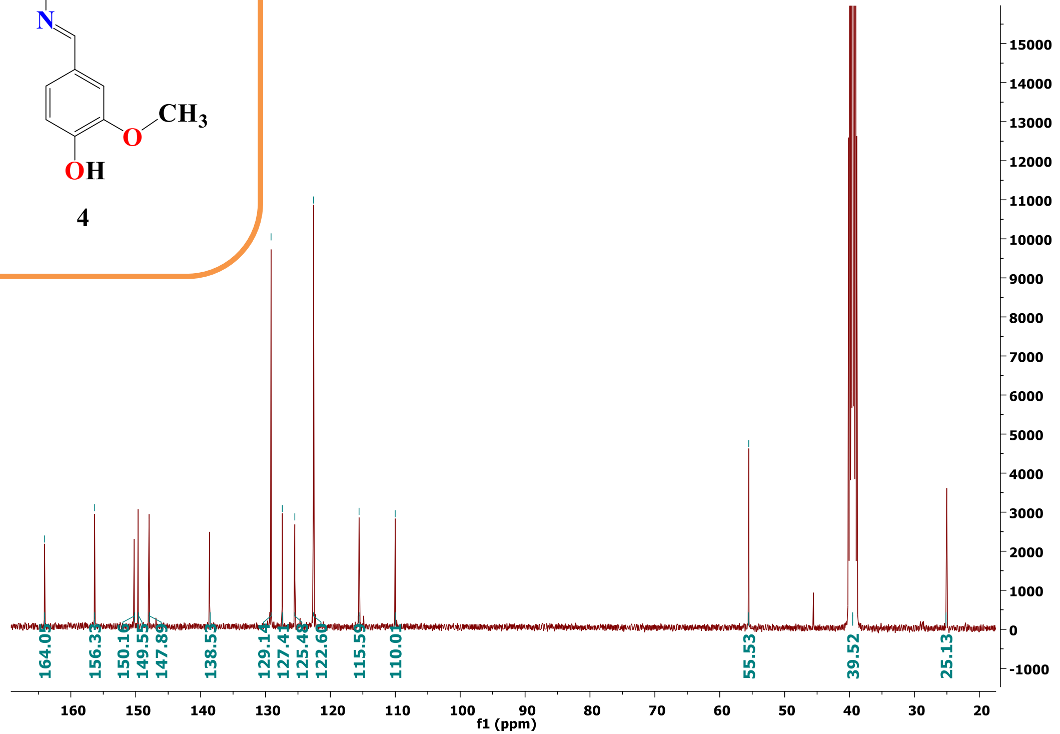

Figure S8: C13NMR spectrum of comp. **4**.

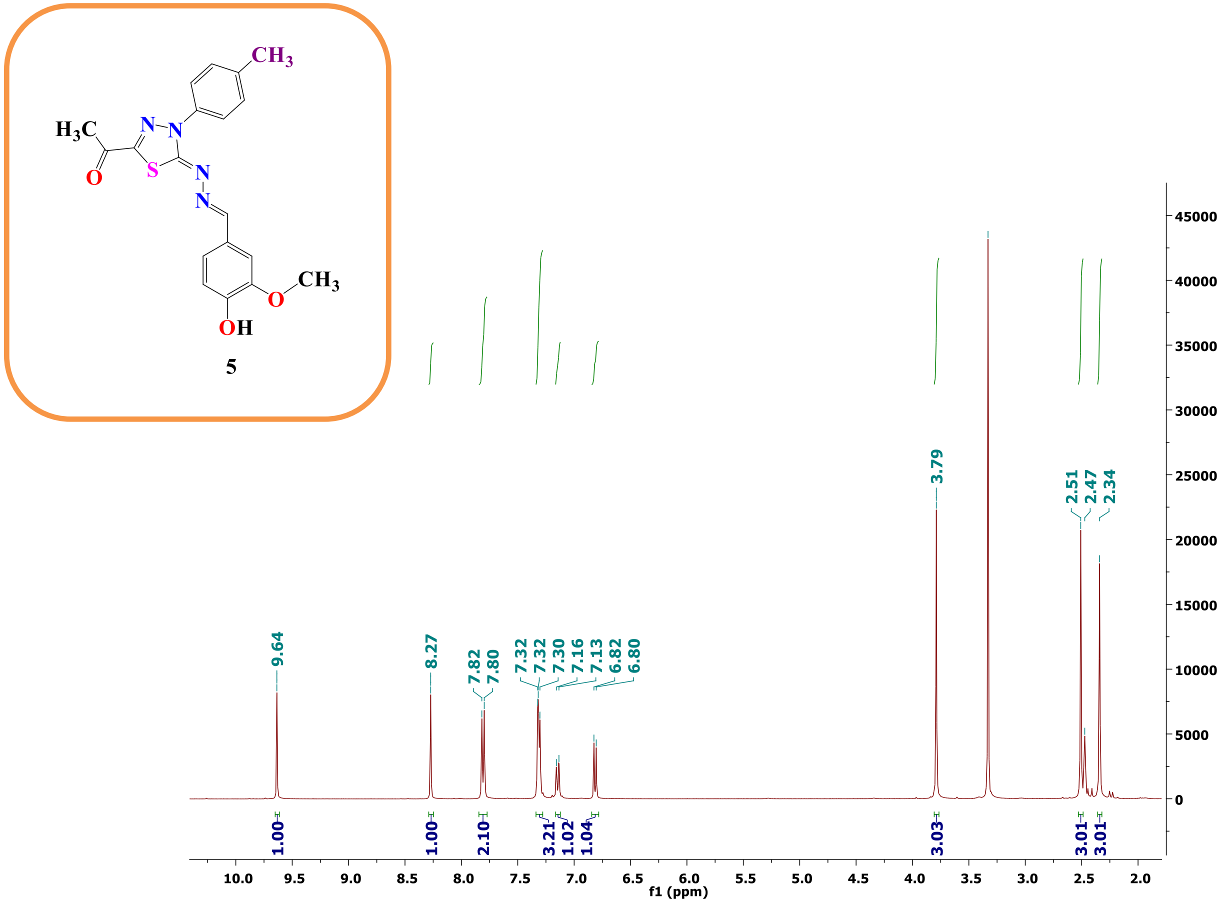

Figure S9: H1NMR spectrum of comp. **5**.

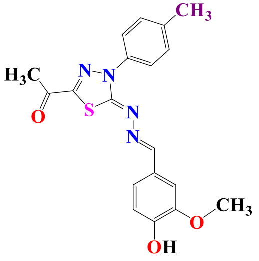

5

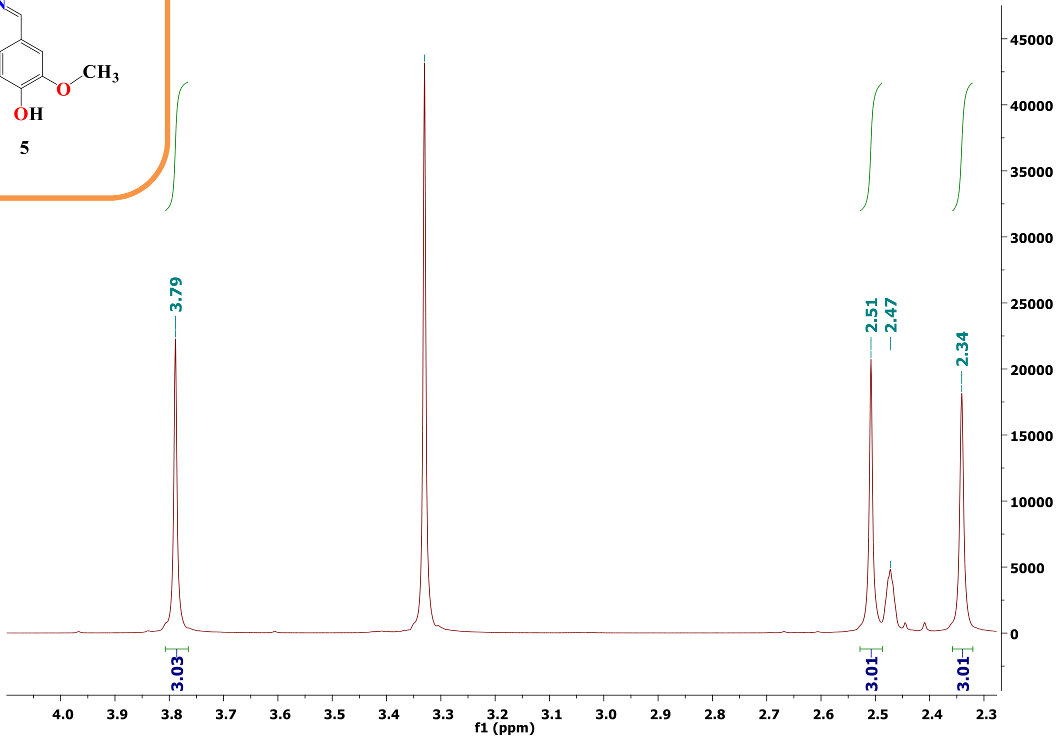

Figure S10: Magnification of H1NMR spectrum of comp. **5**.

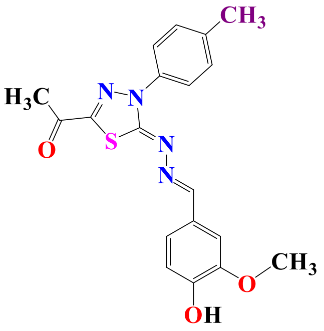

5

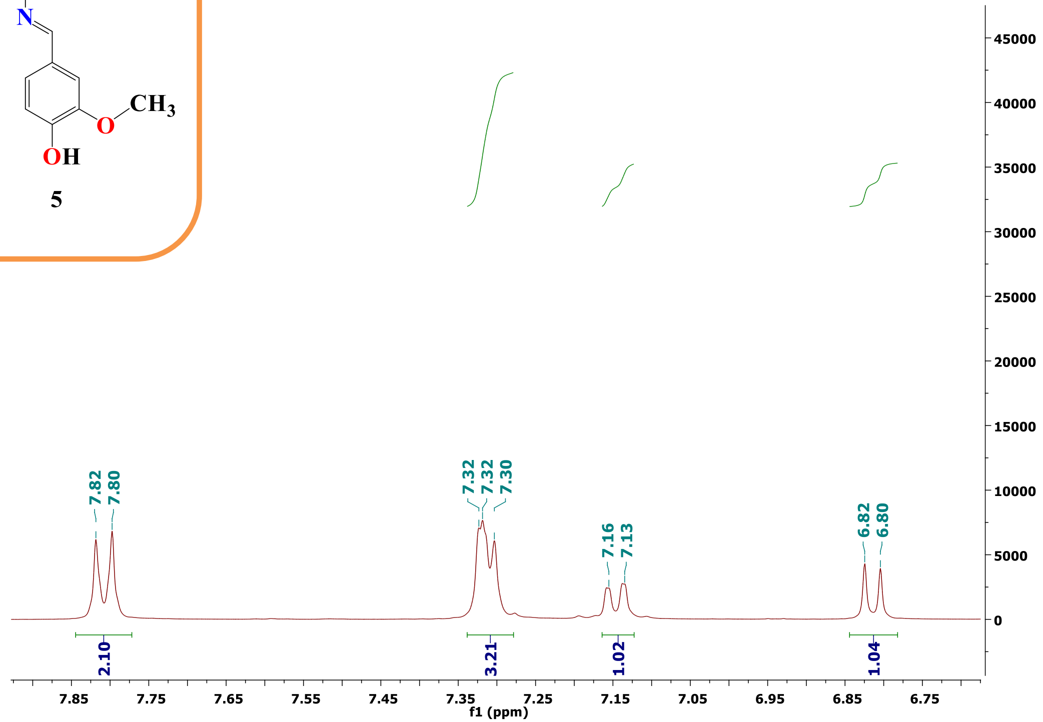

Figure S11: Magnification of H1NMR spectrum of comp. **5**.

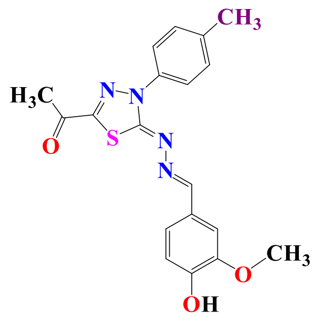

5

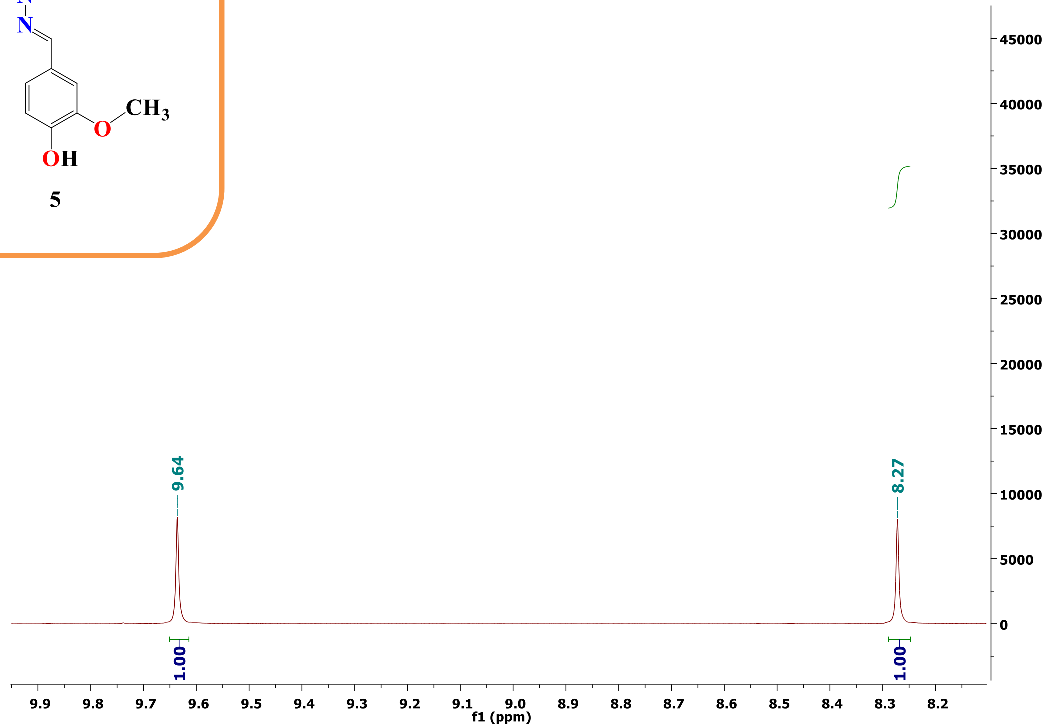

Figure S12: Magnification of H1NMR spectrum of comp. **5**.

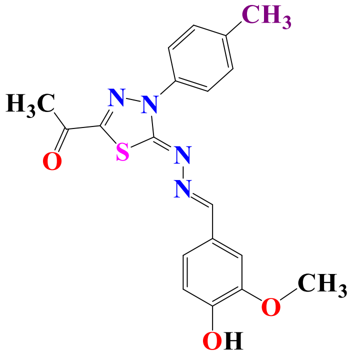

5

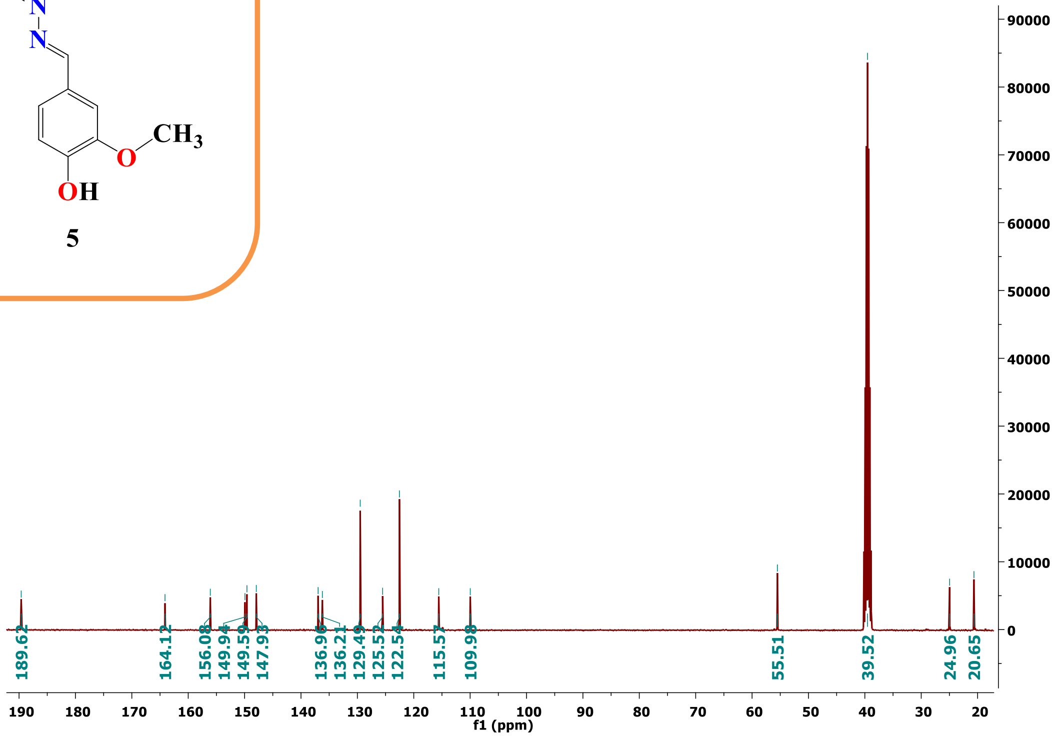

Figure S13: C13NMR spectrum of comp. **5**.

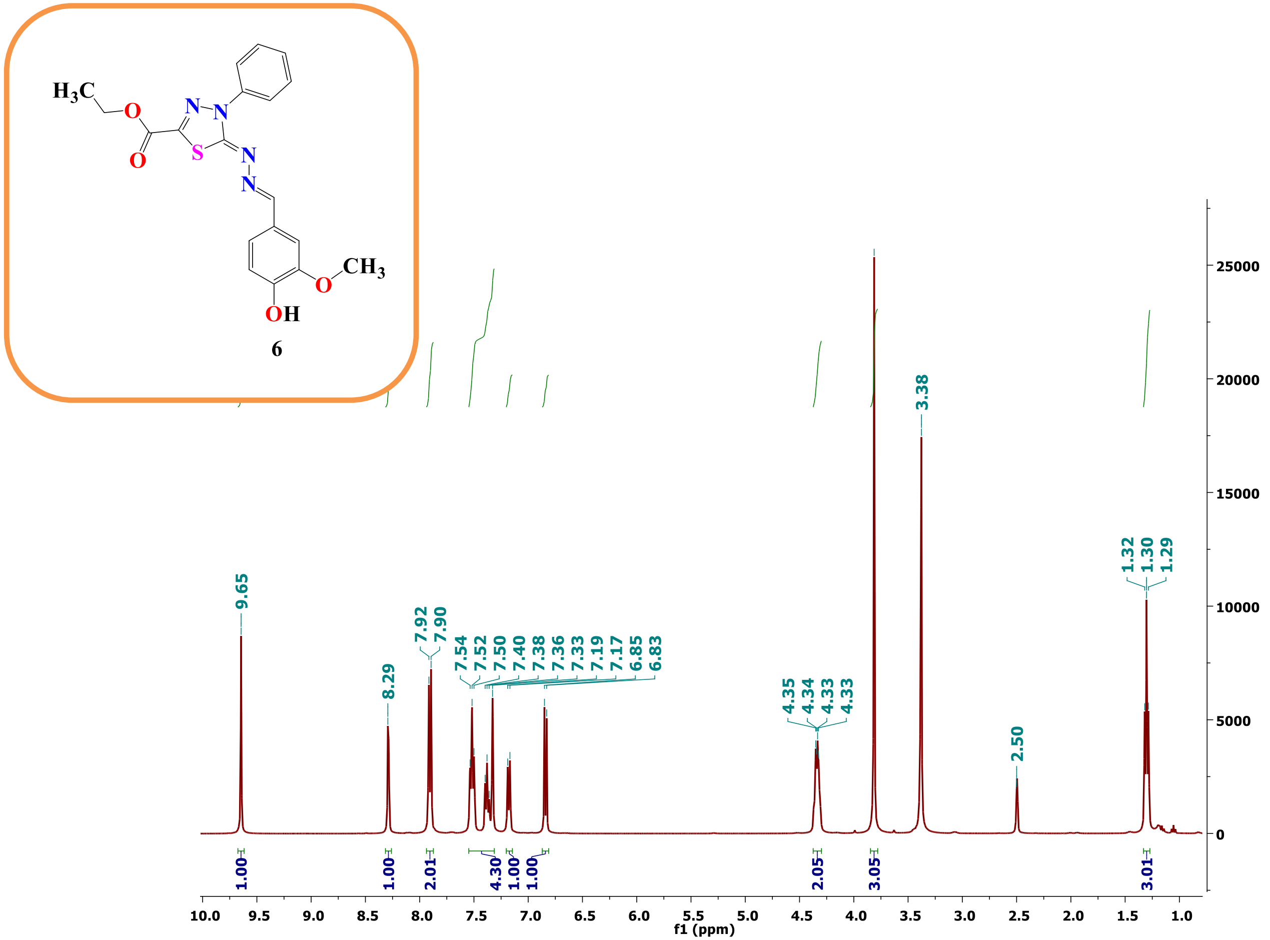

Figure S14: H1NMR spectrum of comp. **6**.

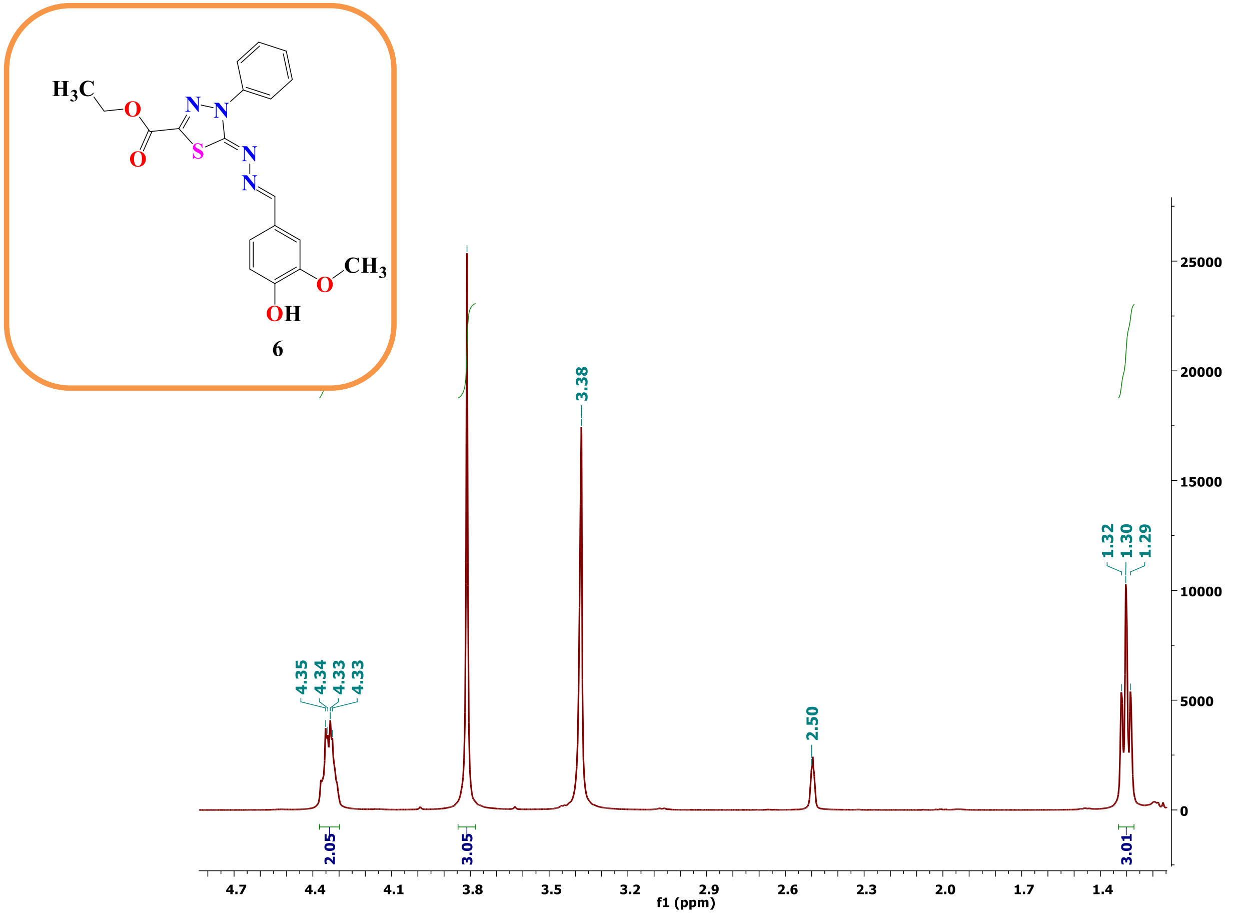

Figure S15: Magnification of H1NMR spectrum of comp. **6**.

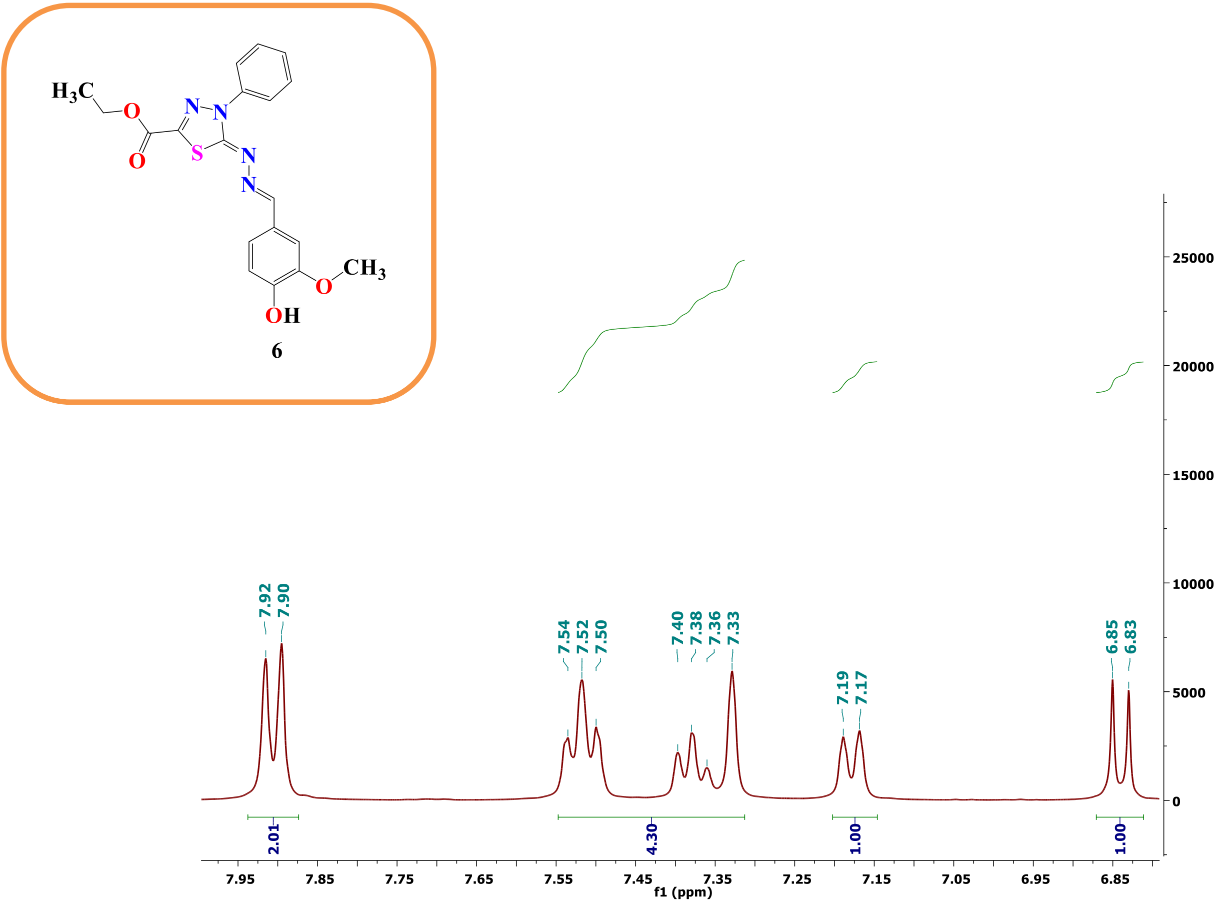

Figure S16: Magnification of H1NMR spectrum of comp. **6**.

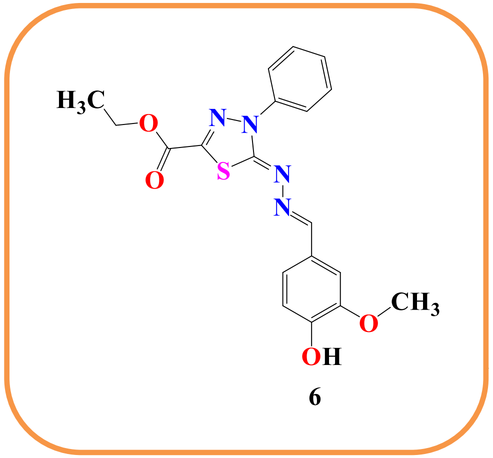

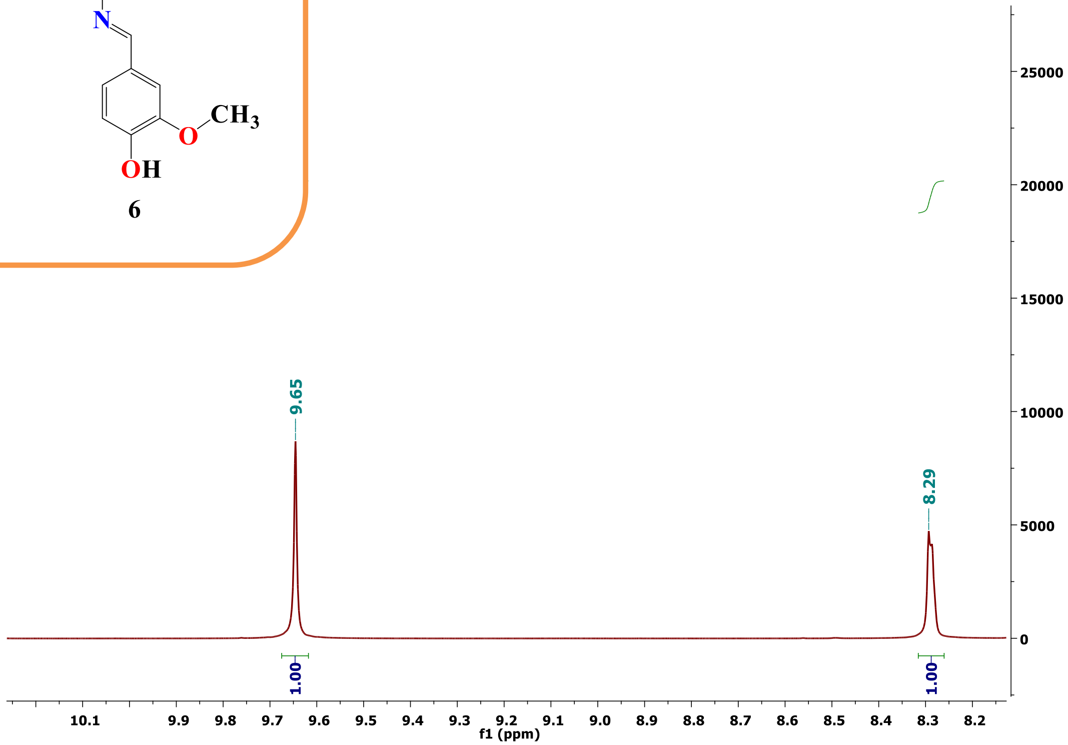

Figure S17: Magnification of H1NMR spectrum of comp. **6**.

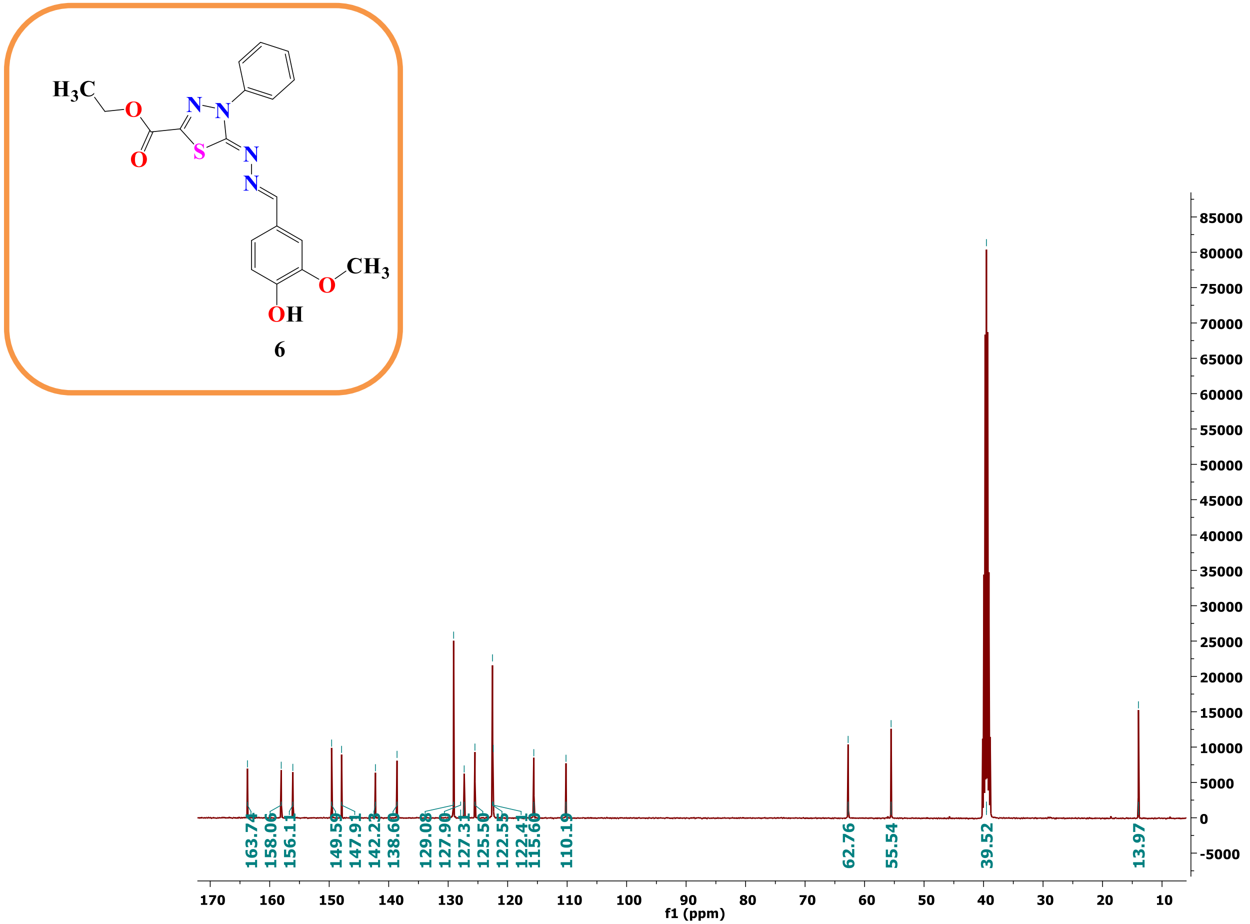

Figure S18: C13NMR spectrum of comp. **6**.

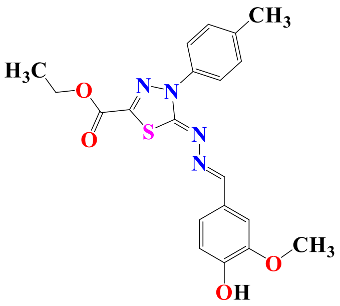

7

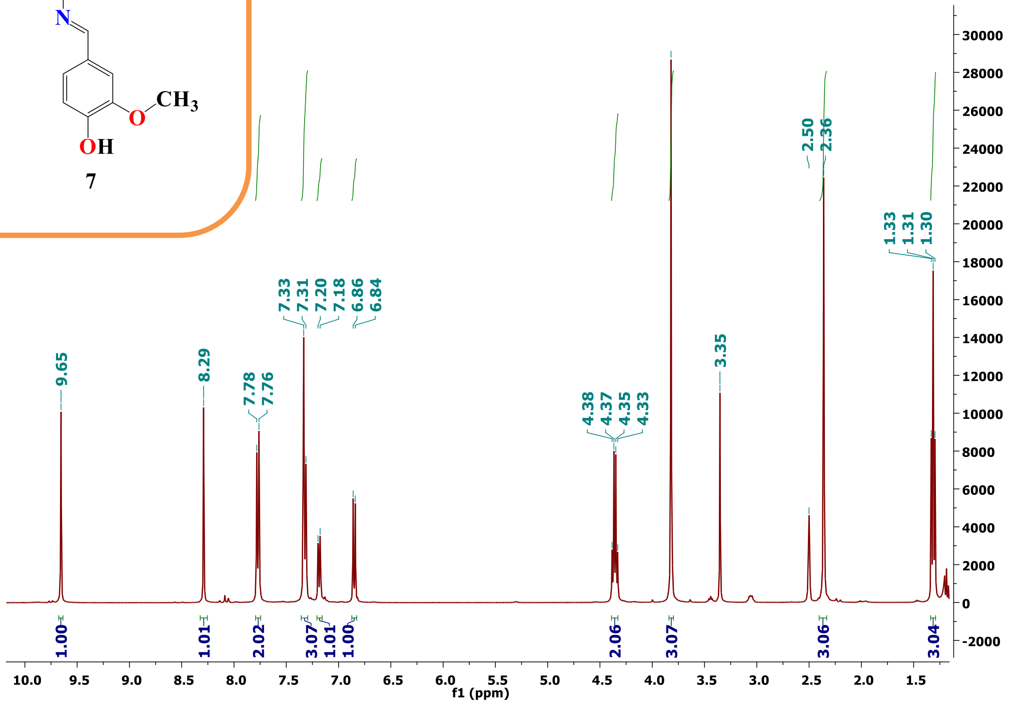

Figure S19: H1NMR spectrum of comp. **7**.

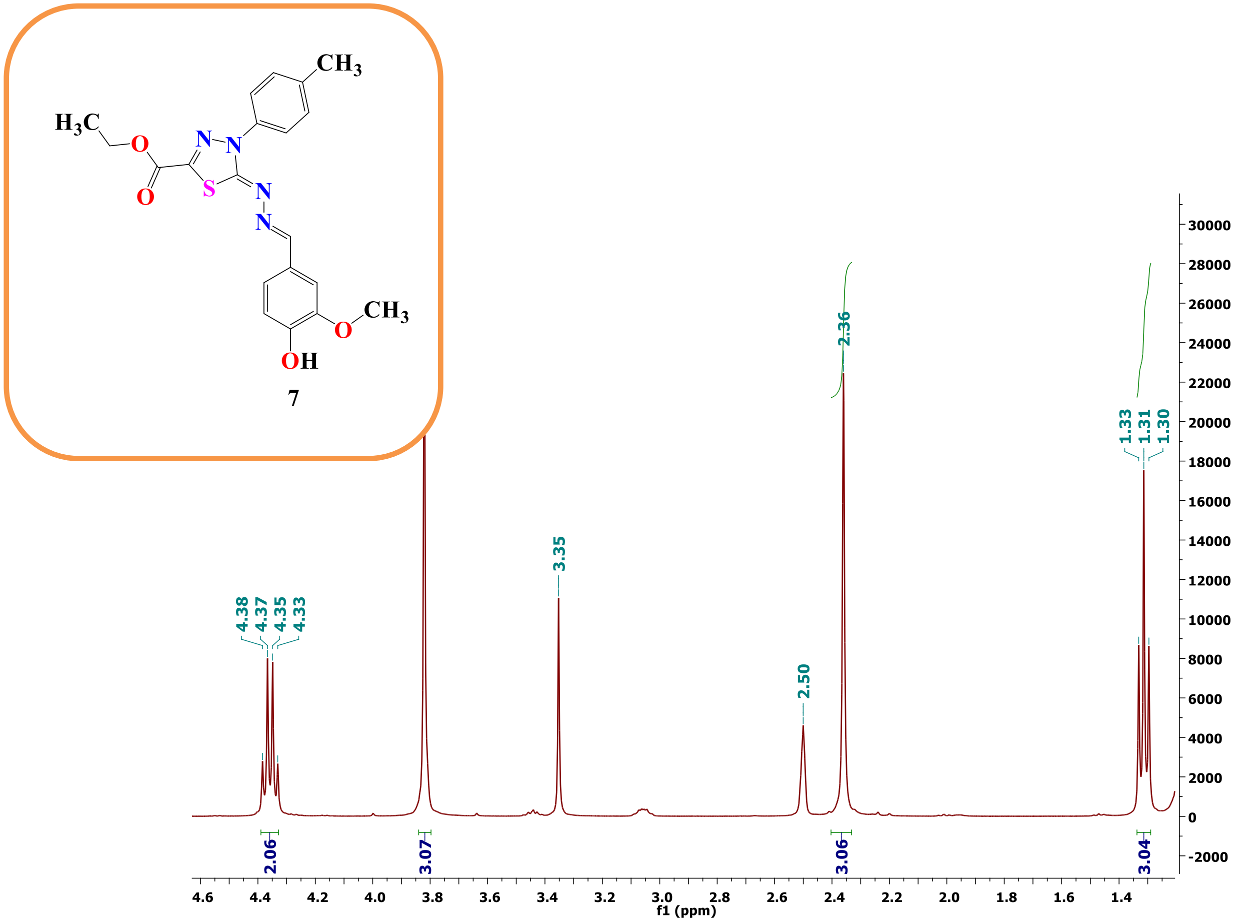

Figure S20: Magnification of H1NMR spectrum of comp. **7**.

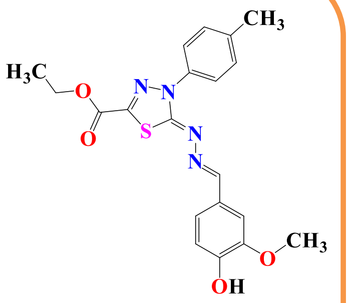

7

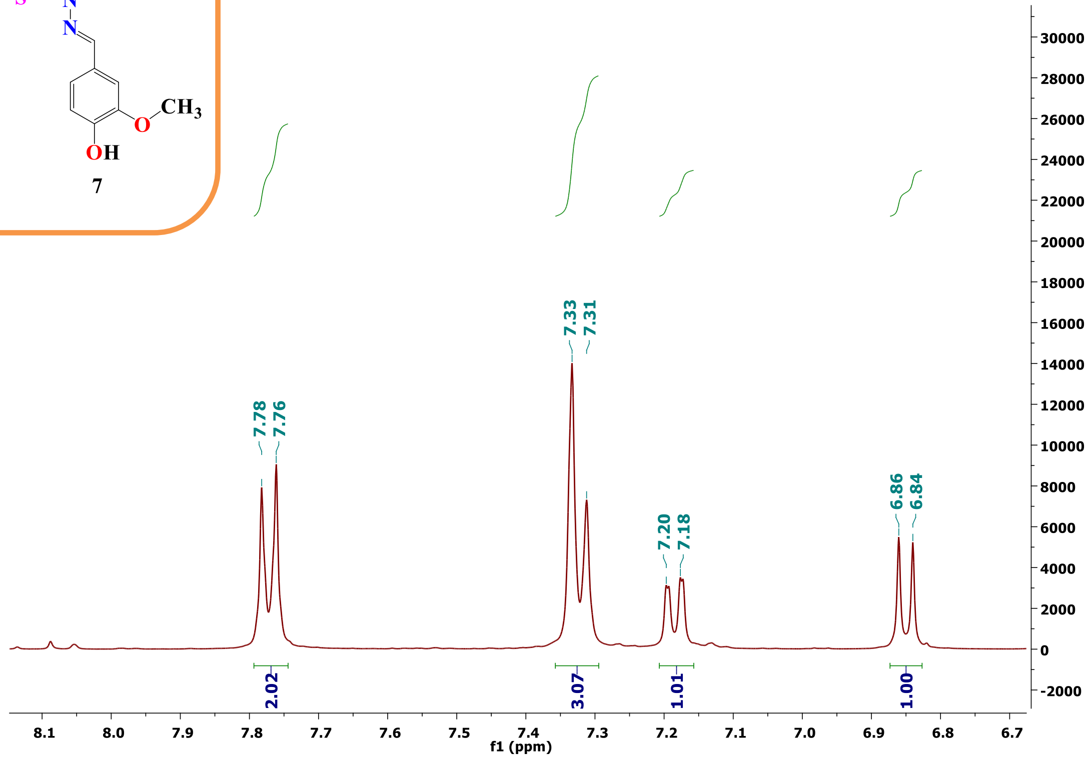

Figure S21: Magnification of H1NMR spectrum of comp. **7**.

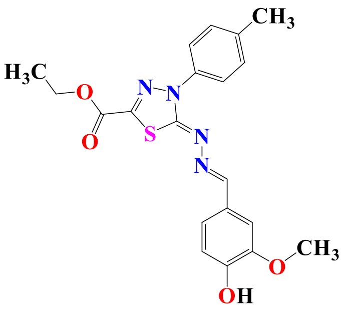

7

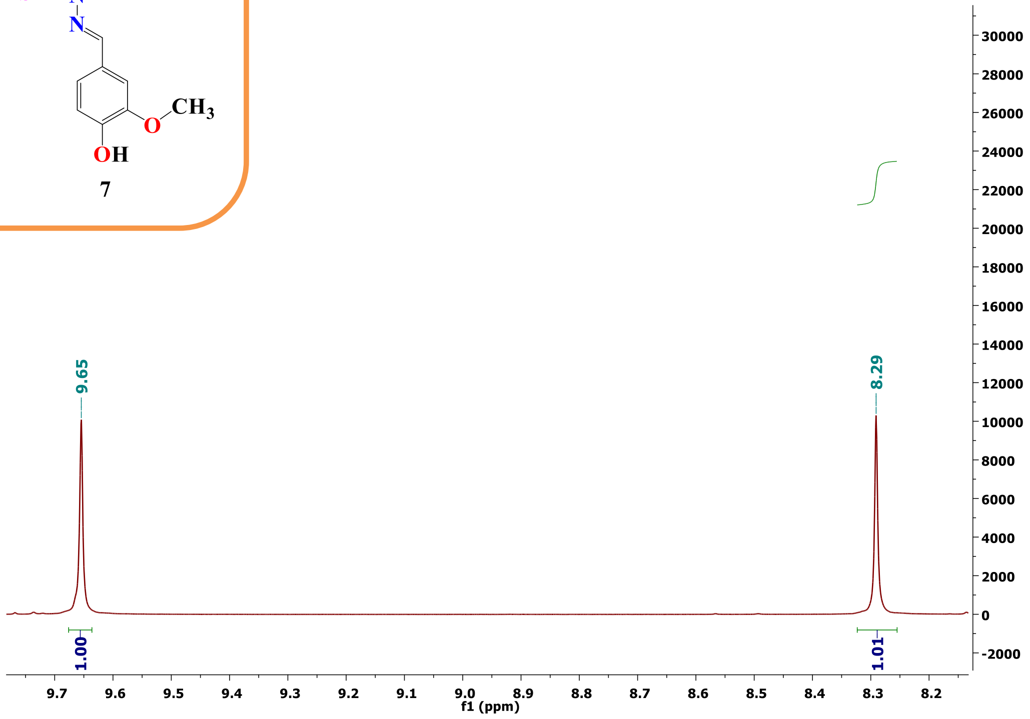

Figure S22: Magnification of H1NMR spectrum of comp. **7**.

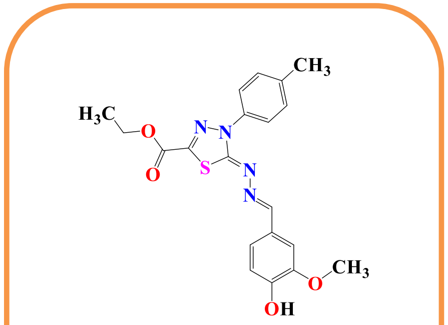

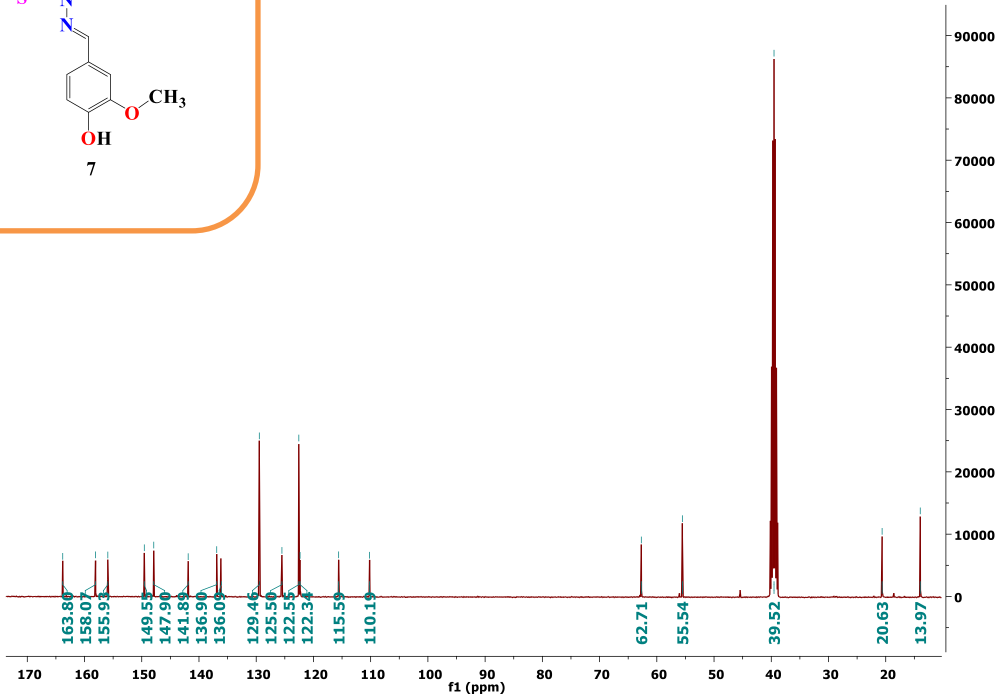

Figure S23: C13NMR spectrum of comp. **7**.

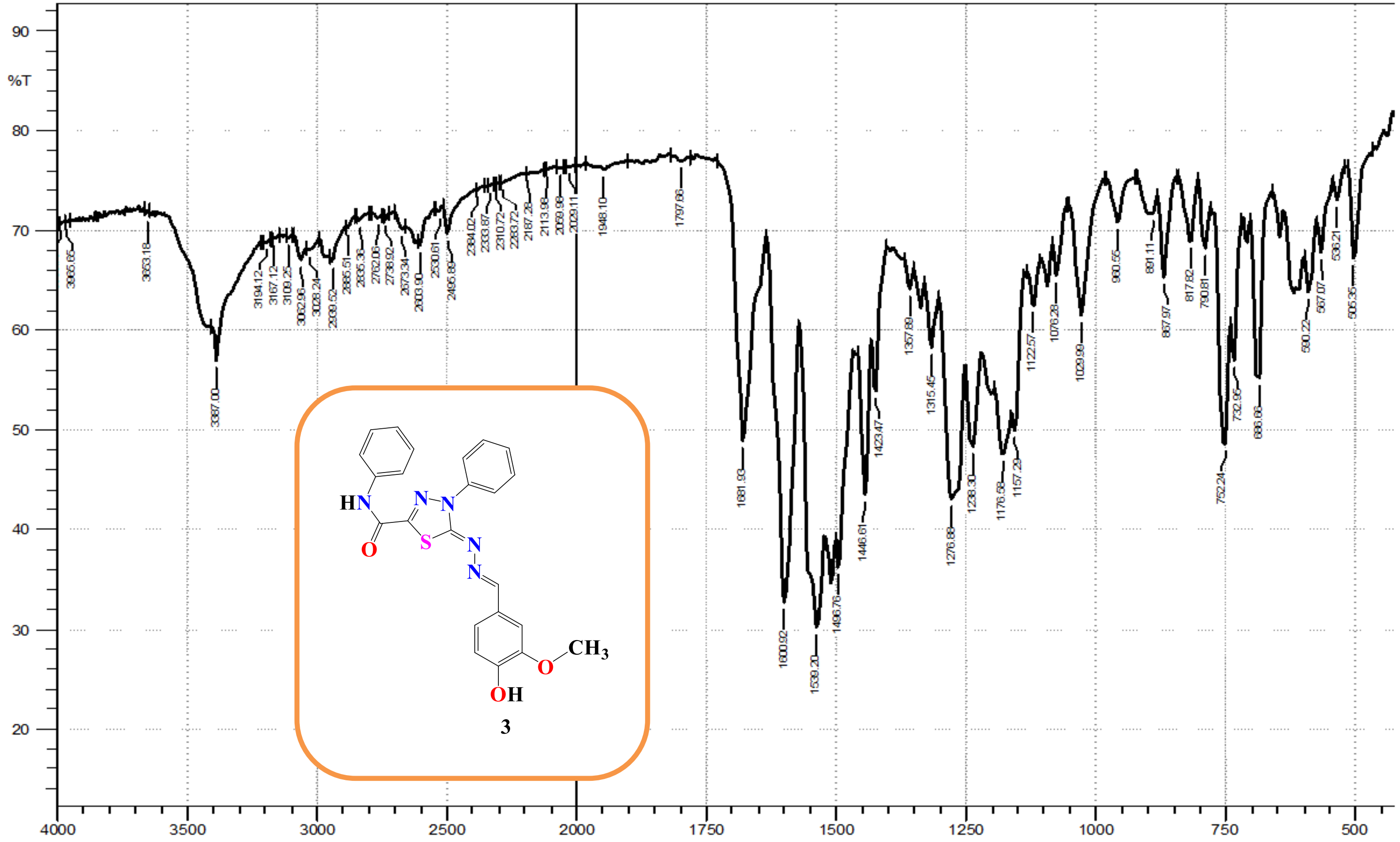

Figure S24: IR of comp. **3**.

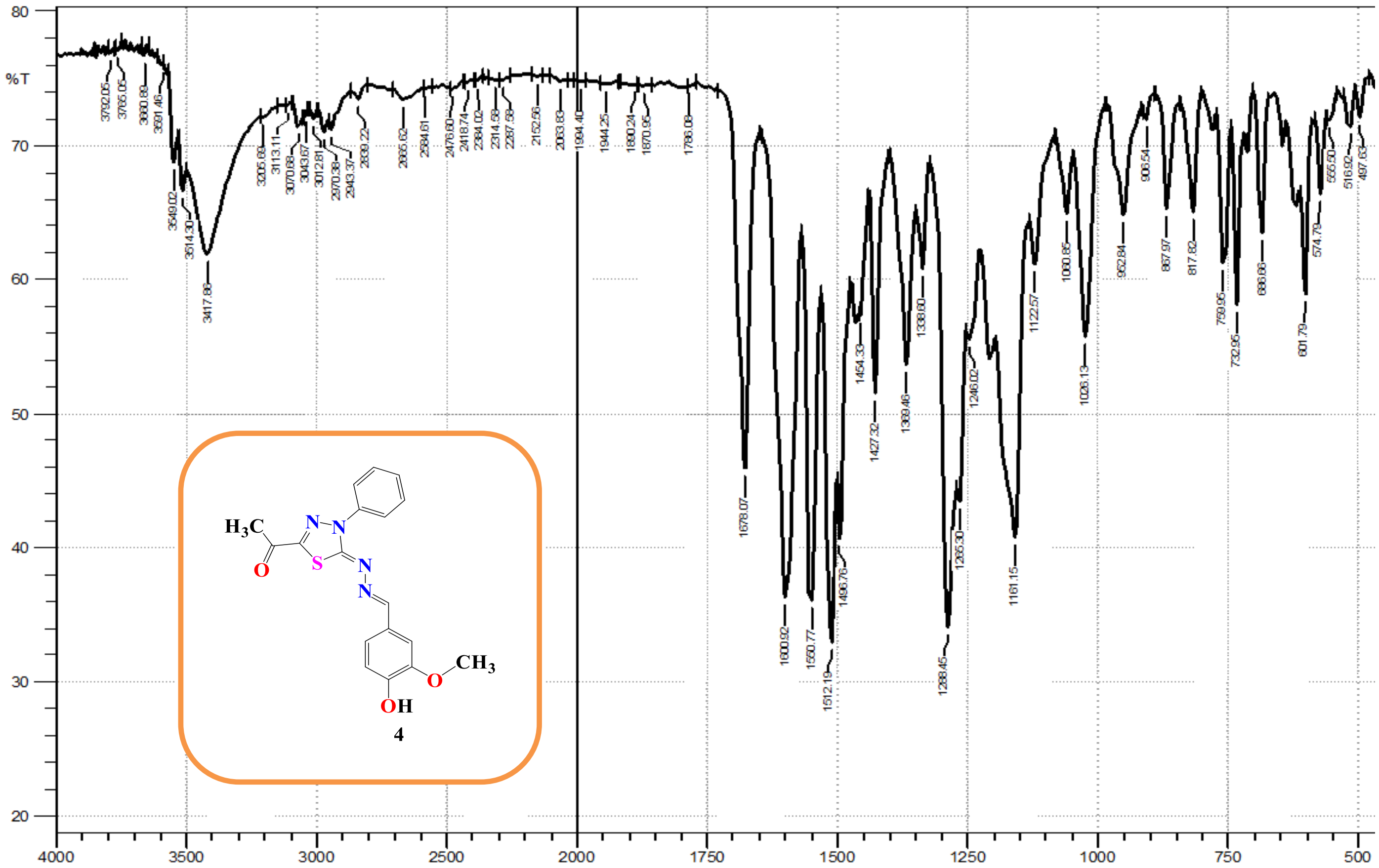

Figure S25: IR of comp. **4**.

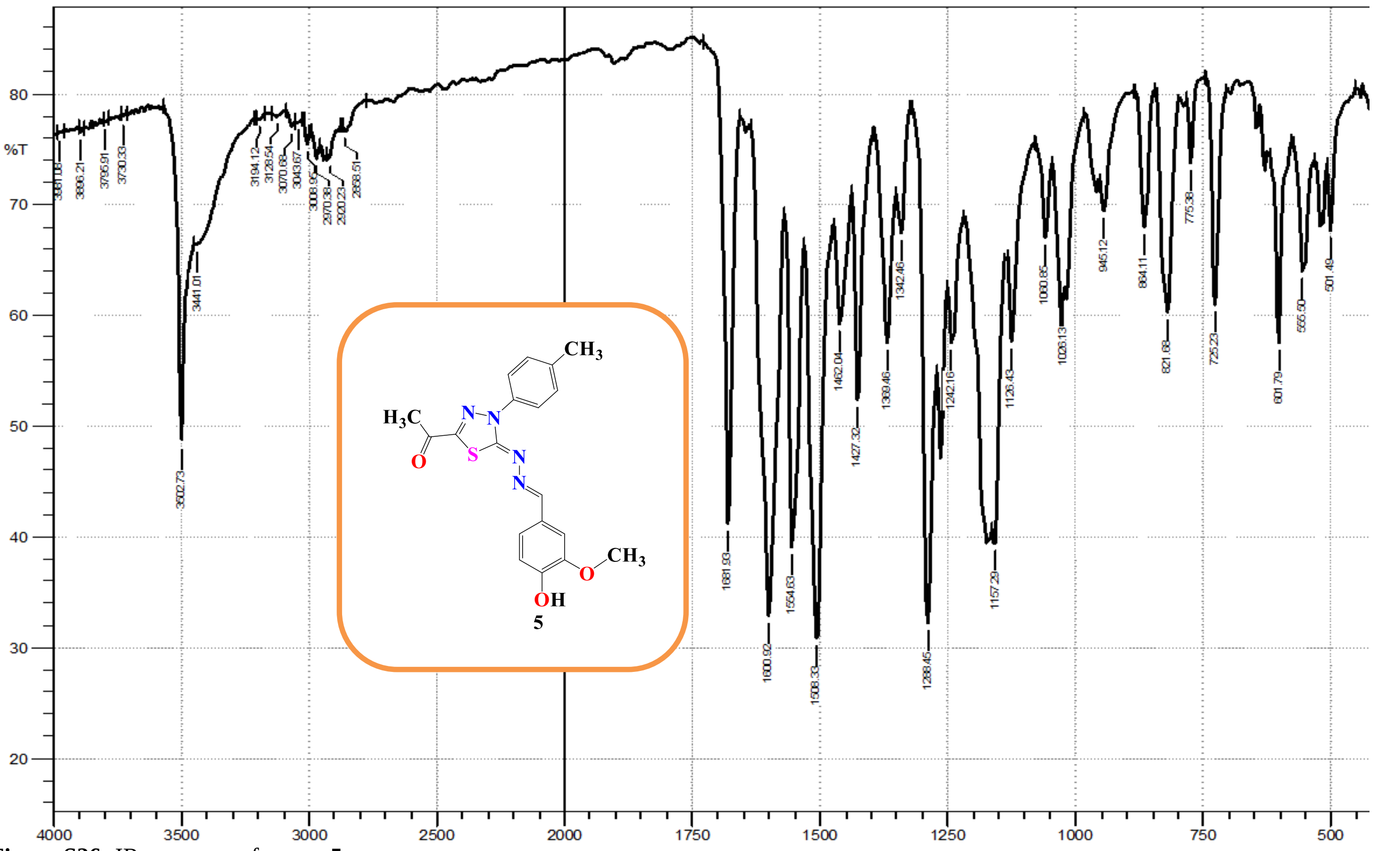

Figure S26: IR spectrum of comp. **5**.

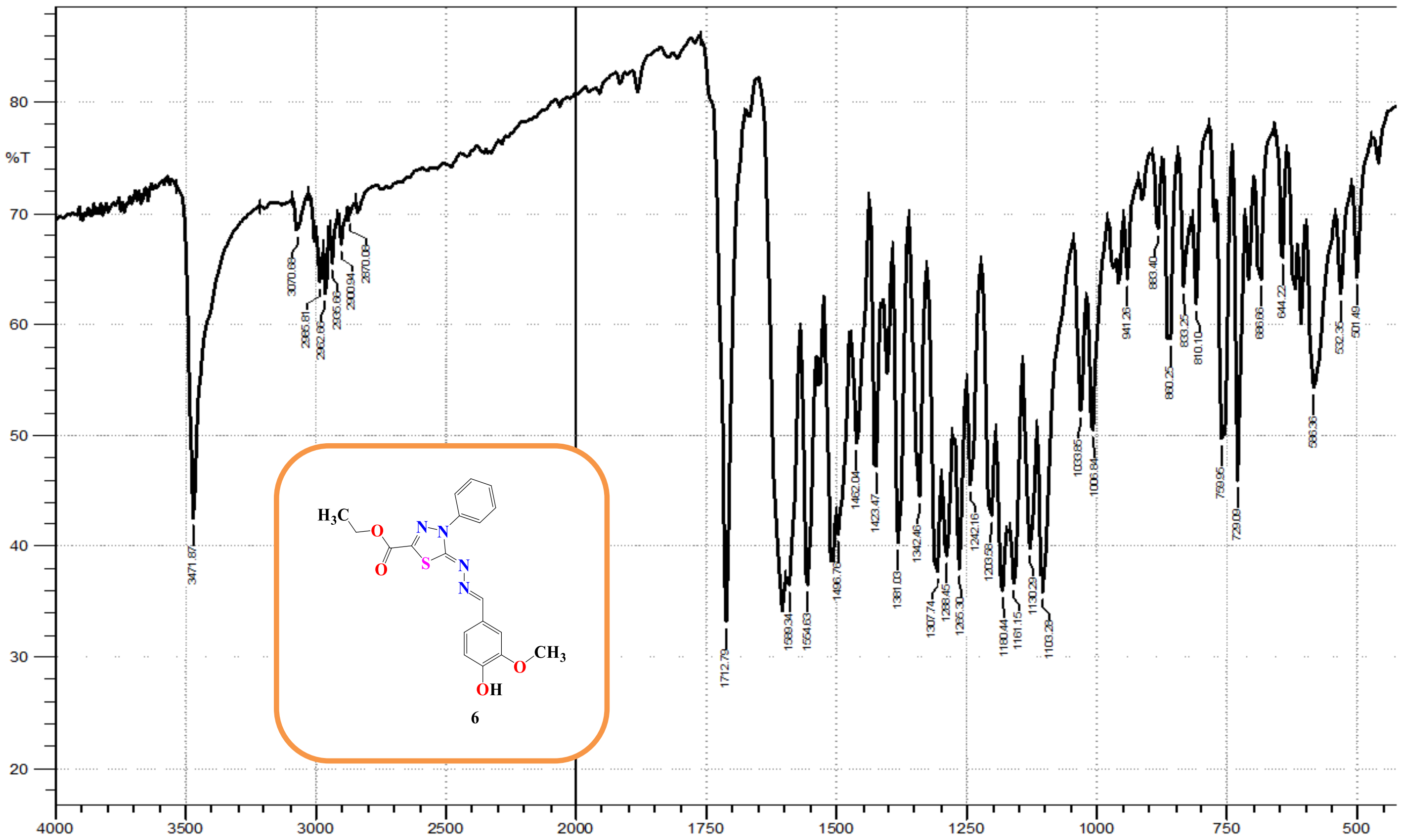

Figure S27: IR of comp. **6**.

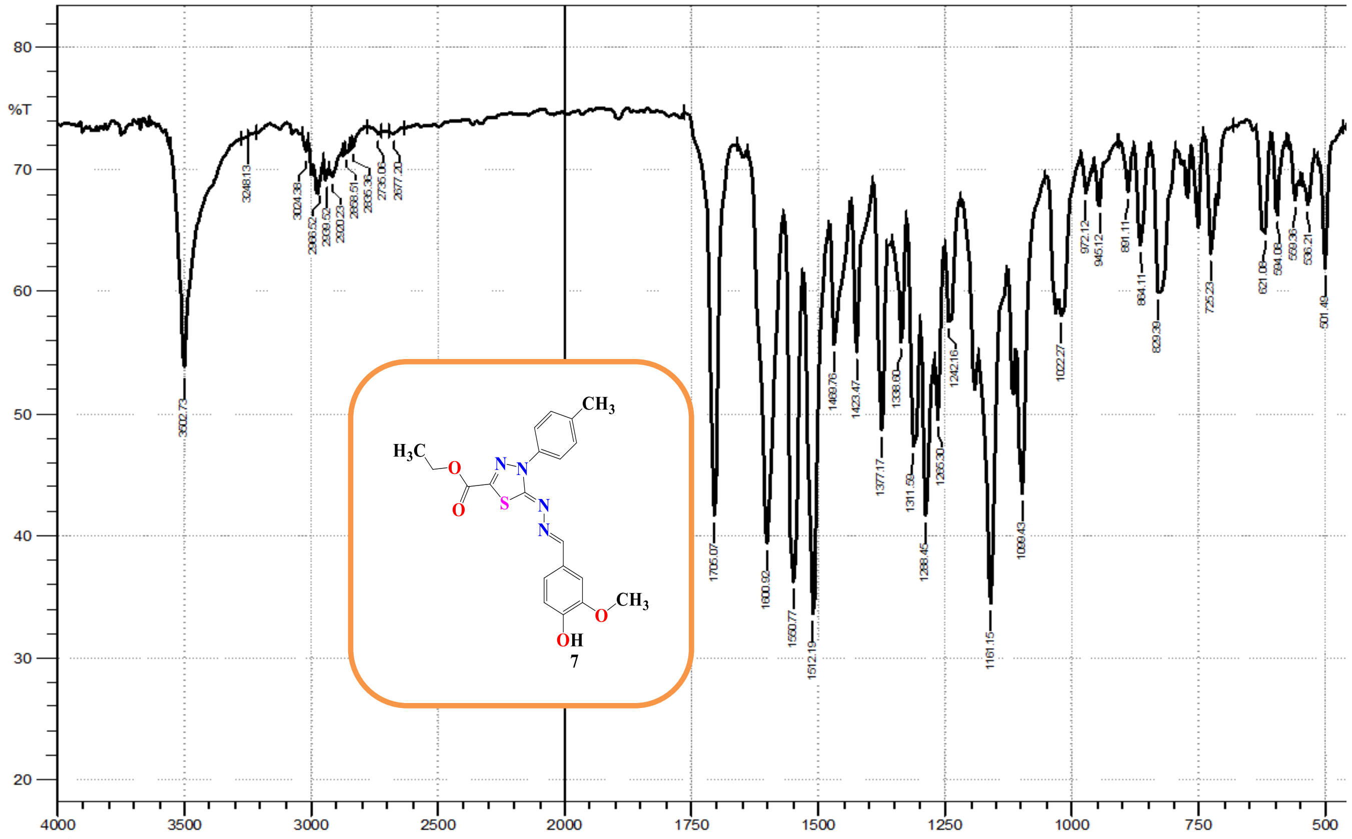

Figure S28**:** IR of comp. **7**.

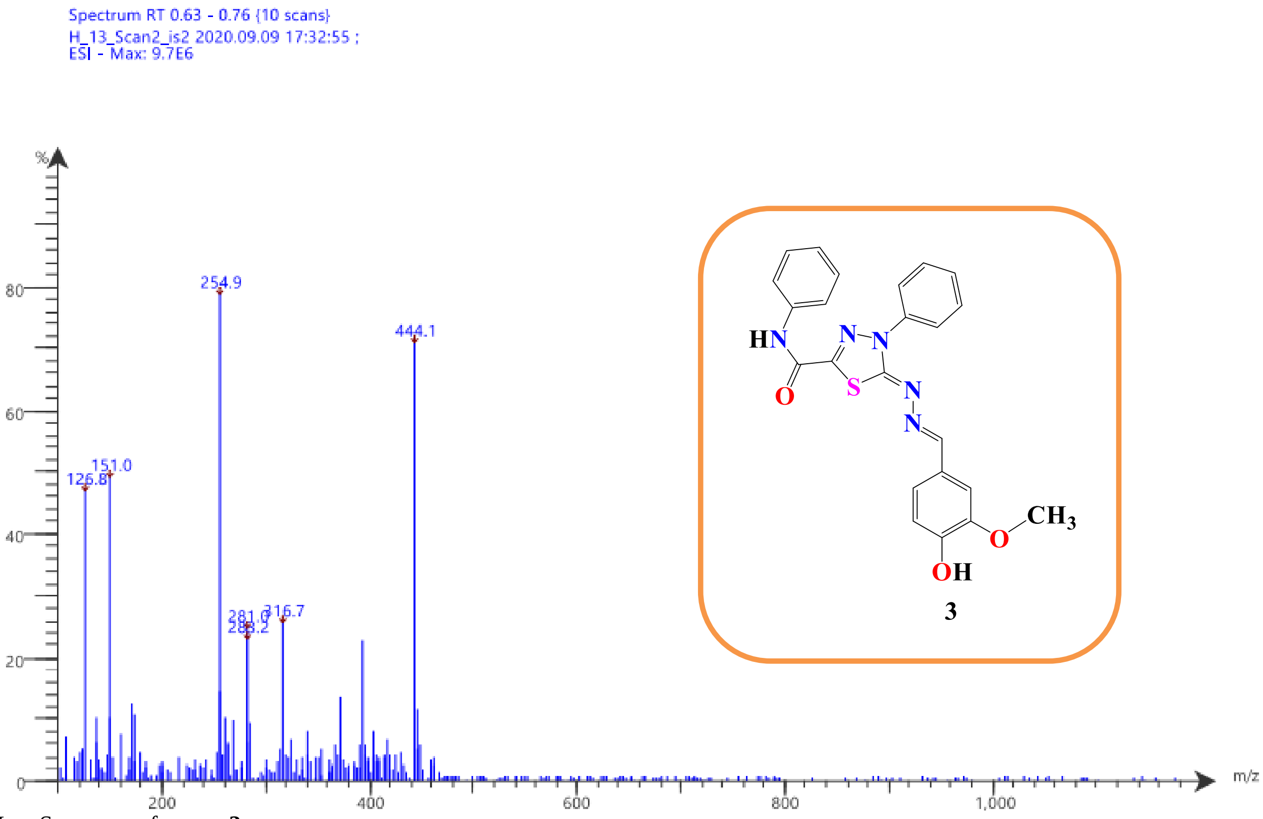

Figure S29: Mass Spectrum of comp. **3**.
